# Ferroptosis in metabolic dysfunction-associated steatotic liver disease

**DOI:** 10.3389/fimmu.2026.1912709

**Published:** 2026-09-17

**Authors:** Stanislav Kotlyarov, Aleksandra Iskrina, Anna Kotlyarova

**Affiliations:** 1 Department of Nursing, Ryazan State Medical University, Ryazan, Russia; 2 Department of Pharmaceutical Technology and Pharmacy Practice Administration, Ryazan State Medical University, Ryazan, Russia

**Keywords:** ferroptosis, inflammation, iron metabolism, lipid peroxidation, metabolic dysfunction-associated steatotic liver disease (MASLD), metabolic dysfunction-associated steatohepatitis (MASH)

## Abstract

Metabolic dysfunction-associated steatotic liver disease (MASLD) is a widespread chronic liver disease that can progress from steatosis to steatohepatitis, fibrosis, cirrhosis, and hepatocellular carcinoma. Its pathogenesis involves lipotoxicity, iron metabolism disorders, and oxidative stress—the same three processes that underlie ferroptosis, an iron-dependent form of regulated cell death mediated by the peroxidation of membrane phospholipids. However, the question of whether ferroptosis is a universal mechanism underlying the progression of MASLD as a whole or a hallmark of a distinct subgroup of patients remains unresolved. Furthermore, no ferroptosis-targeted strategy has yet been tested in a biomarker-selected population. This review addresses precisely this gap. Clinical, transcriptomic, and experimental data allow us to conceptualize hepatic ferroptosis as four interacting molecular modules: expansion of the labile pool of ferrous iron, enrichment of membranes with oxidizable polyunsaturated phospholipids, enzymatic and non-enzymatic lipid peroxidation, and failure of antioxidant defense. An imbalance in these modules leads to the accumulation of phospholipid hydroperoxides, disruption of membrane integrity, and immunogenic hepatocyte death with the release of signals that promote inflammation and fibrogenesis. At the same time, ferroptosis exhibits pronounced cellular and contextual specificity. It exerts a damaging effect on hepatocytes and sinusoidal endothelial cells, enhances inflammation in macrophages, but exerts an antifibrotic effect in activated stellate cells and immunosuppressive effects in lymphocytes. In this regard, known anti-ferroptotic strategies (iron chelation, inhibition of long-chain acyl-coenzyme A (CoA) synthase 4 (ACSL4)-dependent incorporation of polyunsaturated fatty acids, use of lipid radical scavengers and vitamin E, and activation of protective antioxidant programs) retain their diagnostic and therapeutic potential only within the framework of a molecularly and cell-stratified approach.

## 1 Introduction

Metabolic dysfunction-associated steatotic liver disease (MASLD) is a widespread disease (1–7), the epidemiology of which is closely linked to obesity, metabolic syndrome, and diabetes mellitus (8–18). According to the Global Burden of Disease 2023 analysis, the prevalence of MASLD worldwide is steadily increasing, and projections through 2050 indicate a further rise in prevalence (19).

MASLD is a heterogeneous and progressive disease that encompasses both steatosis and steatohepatitis, reflecting a transition from simple lipid accumulation in hepatocytes to the development of inflammation and fibrosis. Furthermore, the disease can progress to liver cirrhosis and is associated with an increased risk of hepatocellular carcinoma (2, 20, 21).

The term “metabolic dysfunction-associated steatotic liver disease” (MASLD) was proposed in 2023 as part of an international, multi-society Delphi consensus under the auspices of leading hepatology, diabetes, and metabolic associations. This new term replaced the old term “nonalcoholic fatty liver disease” (NAFLD) (20, 22–25). This terminological change reflects a shift from a diagnosis based primarily on the exclusion of significant alcohol consumption to a positive definition of the disease based on the presence of hepatic steatosis and cardiometabolic risk factors. The term MASLD is broader than NAFLD, as it incorporates cardiometabolic risk factors (17, 25, 26). Thus, the change in terminology resolved the definitional issue but left the central pathophysiological question unanswered, since the metabolic definition alone does not indicate the effector mechanism that transforms steatosis into steatohepatitis.

Ferroptosis is of particular interest for MASLD/metabolic dysfunction-associated steatohepatitis (MASH) for two reasons. First, it conceptually unites the three central nodes of MASLD pathogenesis into a single causal mechanism of membrane damage, namely iron metabolism disruption, lipid accumulation, and oxidative stress (27, 28). Second, experimental ferroptosis inhibitors such as ferrostatin-1 (Fer-1), liproxstatin-1 (Lip-1), and deferoxamine (DFO), as well as natural vitamin E, consistently reduce the severity of steatohepatitis in several MASLD models, whereas pharmacological induction of ferroptosis (e.g., with the glutathione peroxidase 4 (GPX4) inhibitor Ras Lethal 3 (RSL3)) exacerbates liver damage (27–30). However, ferroptosis should not be regarded as a universal and obligatory mechanism of MASLD progression in all patients. It is more accurate to interpret it as a stratifiable molecular phenotype characteristic of a subgroup of patients with a combination of increased lipid peroxidation, impaired iron metabolism, and weakened anti-ferroptotic defenses. This approach is particularly important for the future translation of anti-ferroptotic strategies, as the efficacy of ferroptosis-targeted therapy will likely depend on the pre-selection of patients with the corresponding tissue or molecular signature. Thus, the unresolved issue is not whether ferroptosis is theoretically possible in MASLD, but rather how to identify the patients and liver cell populations in whom it actually drives disease progression.

Thus, the aim of this review is to discuss the key mechanisms of ferroptosis in the pathogenesis of MASLD. Methodologically, this narrative review included an analysis of available publications on key search terms: MASLD/MASH/NAFLD + ferroptosis; iron metabolism; lipid peroxidation; transcriptomics; liver biopsy, but was not limited to them; as well as on specific key genes and proteins: *ACSL4, GPX4, SLC7A11, TFRC/TFR1, FTH1, FTL, NCOA4, SLC39A14, HMOX1*, ferroptosis suppressor protein 1 (*FSP1*, also known as apoptosis-inducing factor mitochondria-associated 2, *AIFM2*), *GCH1, LPCAT3, ALOX, POR, NOX*, and others. Priority was given to studies involving patients, biopsy and transcriptomic cohorts, as well as experimental models. In addition, the analysis included studies on cholesterol biosynthesis (DHCR7, 7-dehydrocholesterol), mitochondrial and calcium homeostasis, as well as cell-specific ferroptosis in stellate cells, sinusoidal endothelial cells, and immune system cells.

## 2 Pathogenic context of MASLD/MASH

Ferroptosis does not occur in isolation. It is closely linked to the accumulation of toxic lipids, dysfunction of the endoplasmic reticulum, inflammatory signals from the innate immune system, and iron metabolism disorders (29). Together, these processes create the metabolic environment in which regulated cell death occurs.

### 2.1 Diagnostic criteria and the spectrum of disease

According to the Delphi consensus and the 2024 EASL–EASD–EASO clinical guidelines (European Association for the Study of the Liver (EASL), European Association for the Study of Diabetes (EASD), European Association for the Study of Obesity (EASO)), MASLD is diagnosed in the presence of liver steatosis confirmed by histology, imaging, or validated biomarkers, in combination with at least one of the following cardiometabolic risk factors: overweight/obesity, dysglycemia or type 2 diabetes, hypertriglyceridemia, low high-density lipoprotein cholesterol, or hypertension (20, 22–25). It is more accurate to state that a diagnosis of MASLD requires the absence of another obvious dominant cause of steatosis, rather than the absolute exclusion of all potential concomitant factors. The inflammatory form of the disease, characterized by steatosis, ballooning of hepatocytes, and lobular inflammation, is designated as metabolic MASH.

The new classification also identifies the category metabolic dysfunction and alcohol-associated liver disease (MetALD)—a condition in which metabolic dysfunction is combined with alcohol consumption above the threshold acceptable for classic MASLD but below the level typical of alcohol-related liver disease (ALD). MetALD is defined by the presence of hepatic steatosis and at least one cardiometabolic risk factor in combination with alcohol consumption in the range of 140–350 g/week for women and 210–420 g/week for men (20, 22, 31–33). It should be emphasized that in such patients, alcohol itself may contribute to the development of hypertension, hypertriglyceridemia, insulin resistance, and inflammatory liver damage. Therefore, the interpretation of a single cardiometabolic criterion in differentiating between MetALD and ALD should be cautious.

In addition to the cardiometabolic criteria that define MASLD, the likelihood and rate of its progression are determined by a number of modifiable and non-modifiable factors. One of these is a sedentary lifestyle. In patients with established MASLD, time spent sitting is associated with liver fibrosis regardless of total physical activity levels, indicating the independent significance of prolonged physical inactivity, rather than merely a lack of exercise (34). The second axis is biological aging. In the UK Biobank cohort, accelerated biological age predicted the development of newly diagnosed MASLD (35), which is consistent with an age-dependent decline in mitochondrial quality control and antioxidant reserve, factors that determine, among other things, susceptibility to oxidative cell death (36, 37). The third axis is the “gut–liver” interaction, where changes in the composition of the microbiota and spectrum of microbial metabolites modify hepatic lipid metabolism, barrier function, and the tone of the innate immune system (38). This interaction extends to the level of specific cellular death pathways. The translocation of *Bacteroides uniformis (B. uniformis)* from the gut to the liver reduced the severity of advanced MASLD precisely by suppressing hepatocyte ferroptosis, mediated by propionate secretion (39). Thus, lifestyle, age, and the microbiota influence not only the lipid load on the liver but also the threshold at which a hepatocyte enters a regulated cell death pathway. However, the mechanisms through which these factors regulate specific cell death pathways have not yet been studied.

### 2.2 Lipotoxicity, endoplasmic reticulum stress and innate immune activation

The pathogenesis of MASLD/MASH is a multifactorial process in which lipotoxicity, insulin resistance, impaired mitochondrial and peroxisomal fatty acid oxidation, endoplasmic reticulum stress, oxidative stress, dysfunction of the innate immune system, and remodeling of intercellular interactions in the liver play a central role (40–42). Lipotoxicity is caused not so much by the accumulation of neutral triglycerides in lipid droplets as by the formation of toxic lipid species, such as saturated fatty acids, free cholesterol, ceramides, diacylglycerols, lysophosphatidylcholine, and oxidized phospholipids (40–44). These molecules activate endoplasmic reticulum stress via the inositol-requiring enzyme 1α (IRE1α), protein kinase RNA-like ER kinase (PERK), and activating transcription factor 6α (ATF6α) (41, 43, 44), disrupt mitochondrial function, enhance the production of reactive oxygen species, and trigger inflammatory signals of the innate immune system, including the activation of nuclear factor kappa B (NF-κB) and the NOD-, LRR- and pyrin domain-containing protein 3 (NLRP3) inflammasome (41, 42). The result is hepatocyte death, which occurs via apoptosis (41, 43, 44), necroptosis, pyroptosis, and ferroptosis (42, 44), which collectively leads to disease progression.

One of the key mechanisms of MASLD/MASH pathogenesis is the mutual reinforcement of mitochondrial dysfunction and endoplasmic reticulum (ER) stress via mitochondria-associated membranes (MAM)—specialized membrane contact zones between these organelles that facilitate cross-regulation via lipid and calcium exchange. Pathological remodeling of MAM plays a decisive role in disease progression (42, 43, 45, 46). Another key mechanism in the pathogenesis of MASLD/MASH involves inflammatory signals from the innate immune system, triggered by lipotoxicity and the release of damage-associated molecular patterns (DAMPs) (such as adenosine triphosphate (ATP), high-mobility group box 1 (HMGB1), mitochondrial DNA, and extracellular RNAs) from damaged hepatocytes. The binding of DAMPs to Toll-like receptors (TLRs) (such as TLR2, TLR4, TLR9, and TLR3) activates NF-κB and triggers the assembly of the NLRP3 inflammasome. This results in the secretion of pro-inflammatory cytokines (interleukin-1β (IL-1β), interleukin-18 (IL-18), tumor necrosis factor α (TNF-α), interleukin-6 (IL-6)) and chemokines (C-C motif chemokine ligand 2 (CCL2), C-X-C motif chemokine ligand 1 (CXCL1)), which recruit and activate monocytes/macrophages and neutrophils. The polarization of macrophages into a pro-inflammatory phenotype (M1) and the formation of neutrophil extracellular traps (NETs) create and sustain a chronic inflammatory environment that promotes the progression of steatosis to steatohepatitis and fibrosis (47–50).

### 2.3 Disturbed iron homeostasis

Iron metabolism disorders, often manifesting as iron overload syndrome, are one of the key mechanisms in the pathogenesis of MASLD and MASH (51, 52). Intrahepatic accumulation of excess iron via the Fenton reaction leads to the generation of highly reactive hydroxyl radicals and oxidative stress (52, 53). This directly damages hepatocytes and induces ferroptosis (27). The DAMPs released during this process activate pro-inflammatory cascades and profibrogenic signaling pathways, contributing to the transformation of stellate cells and an accelerated transition from steatosis to steatohepatitis (27, 52, 53). However, clinical associations between iron, inflammation, and fibrosis do not in themselves constitute evidence of ferroptosis. The set of criteria required for such a conclusion is listed below in the section on the iron module.

## 3 Role of ferroptosis in the pathogenesis of MASLD

Ferroptosis is a regulated form of cell death characterized by necrotic morphology. Its mechanism differs fundamentally from both apoptosis and other necrotic pathways—necroptosis and pyroptosis (54, 55). A key biochemical feature of ferroptosis is the iron-dependent accumulation of toxic concentrations of lipid hydroperoxides. These hydroperoxides are formed as a result of the oxidation of polyunsaturated fatty acids (PUFAs) within membrane phospholipids, which can occur via both non-enzymatic (free-radical chain oxidation initiated by the Fenton reaction) and enzymatic pathways, catalyzed by lipoxygenases (LOXs) (54, 56). Under normal conditions, this oxidation is effectively suppressed by antioxidant systems. However, when these systems fail, hydroperoxides accumulate to lethal concentrations. A central link in the pathogenesis of ferroptosis is precisely this disruption of antioxidant defense, caused mainly by dysfunction of the cystine/glutamate antiporter system Xc^-^ (Xc^-^ system)/reduced glutathione (GSH)/GPX4 axis, specifically by inhibition of GPX4 activity and/or depletion of its cofactor—GSH (54, 56). Such dysregulation leads to an uncontrolled chain reaction of lipid peroxidation, disruption of membrane integrity, and subsequent cell lysis (**Figure 1**).

**Figure 1:**
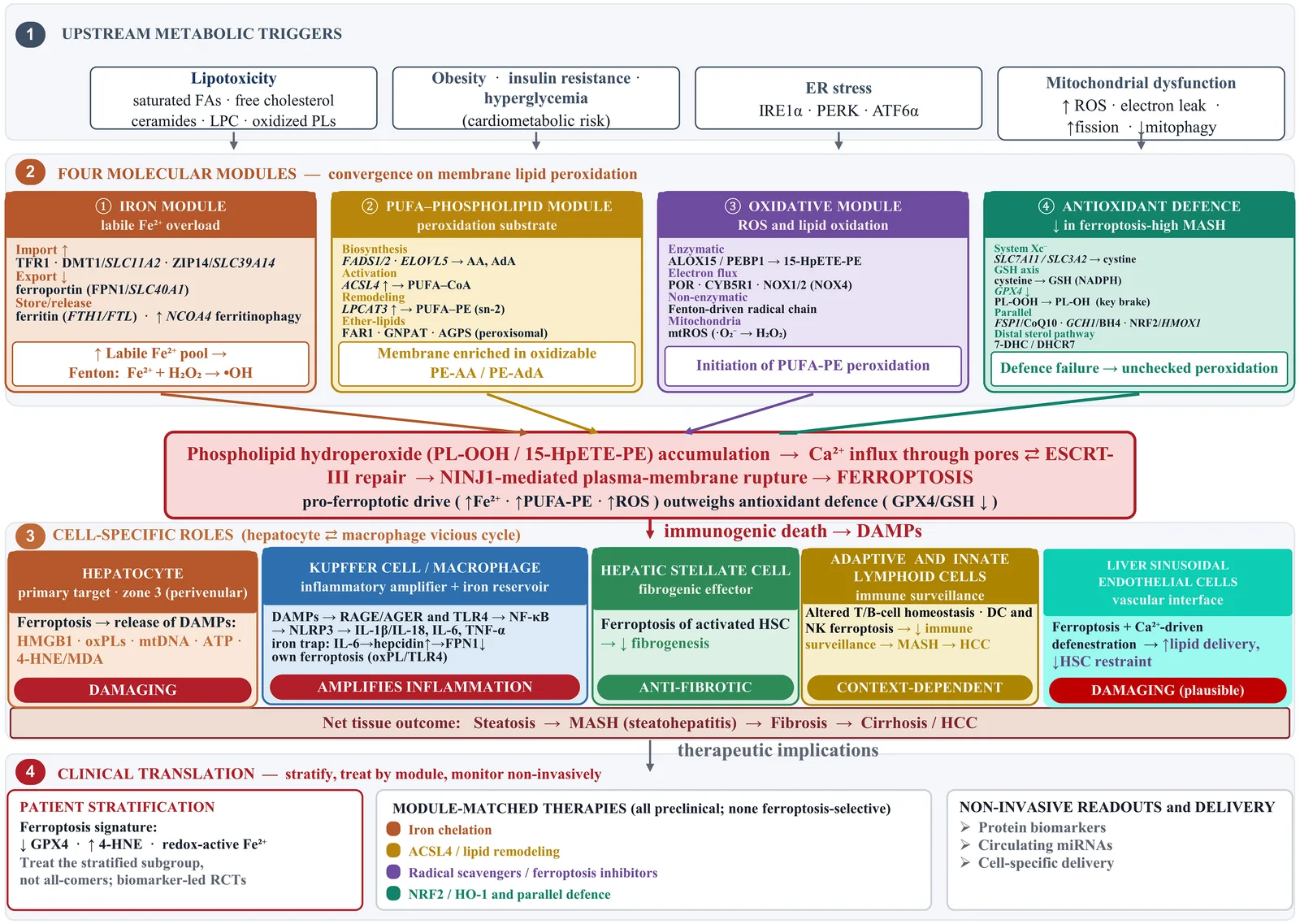
Integrated scheme of ferroptosis in MASLD/MASH: from metabolic triggers and molecular modules to cell-specific outcomes and clinical translation The image is organised as four horizontal tiers, numbered 1–4 in the left margin and read from top to bottom. They correspond to the sections on the pathogenic context of MASLD/MASH, the molecular mechanisms of ferroptosis, cell specificity and intercellular loops, and therapeutic prospects. Tier 1 — Upstream metabolic triggers. Four boxes summarise the metabolic environment in which hepatic ferroptosis arises: lipotoxicity (saturated fatty acids, free cholesterol, ceramides, lysophosphatidylcholine, oxidised phospholipids); obesity, insulin resistance and hyperglycaemia as the defining cardiometabolic context; endoplasmic reticulum stress transduced by IRE1α, PERK and ATF6α; and mitochondrial dysfunction with increased reactive oxygen species production, electron leak, predominance of fission over fusion, and suppressed mitophagy. Four downward arrows denote convergence of these triggers on the molecular modules of tier 2. Tier 2 — Four molecular modules converging on membrane lipid peroxidation. ① Iron module: increased import, reduced export and mobilisation of storage iron expand the labile Fe²^+^ pool and drive the Fenton reaction. ② PUFA–phospholipid module: biosynthesis and elongation of ω-6 fatty acids, ACSL4-dependent activation to PUFA–CoA, LPCAT3-dependent esterification at the sn-2 position, and the peroxisomal ether-lipid branch generate membranes enriched in oxidisable PE-AA and PE-AdA. ③ Oxidative module: enzymatic oxidation by ALOX15 in complex with PEBP1, electron flux through POR, CYB5R1 and NOX1/NOX2, non-enzymatic Fenton-driven radical chain propagation, and mitochondrial reactive oxygen species initiate peroxidation. ④ Antioxidant defence: cystine import through system Xc^-^, glutathione synthesis, GPX4-mediated reduction of phospholipid hydroperoxides to the corresponding alcohols as the key brake, the parallel FSP1/CoQ10, GCH1/BH4 and NRF2/HMOX1 circuits, and the distal cholesterol biosynthetic pathway, in which 7-dehydrocholesterol (7-DHC) acts as an endogenous membrane-embedded radical-trapping antioxidant while 7-dehydrocholesterol reductase (DHCR7), by converting it to cholesterol, acts as a pro-ferroptotic factor. These circuits are attenuated in the ferroptosis-high MASH subgroup. The upper line gives the chain itself: accumulation of phospholipid hydroperoxides (PL-OOH, 15-HpETE-PE) → Ca²^+^ influx through nascent plasma-membrane pores, counterbalanced by ESCRT-III-dependent membrane repair, → NINJ1-mediated plasma-membrane rupture → ferroptosis. The lower line states the condition under which this occurs: ferroptosis ensues when the pro-ferroptotic drive (↑Fe²^+^, ↑PUFA-PE, ↑ROS) outweighs antioxidant defence (↓GPX4/GSH). The vertical arrow to tier 3 is labelled “immunogenic death → DAMPs” and denotes the immunogenic nature of this death. Tier 3 — Cell-specific outcomes. The same execution mechanism has different consequences in different hepatic cell populations, indicated by the colour coding of the columns and by the status bar beneath each cell type. Hepatocytes (the primary target, accentuated in perivenular zone 3) undergo immunogenic death with release of HMGB1, oxidised phospholipids, mitochondrial DNA, ATP and 4-HNE/MDA adducts — status “damaging”. Kupffer cells and monocyte-derived macrophages respond through RAGE/AGER and TLR4 → NF-κB → NLRP3 → IL-1β/IL-18, IL-6 and TNF-α, act as an intrahepatic iron trap (IL-6 → hepcidin ↑ → FPN1 ↓) and undergo ferroptosis themselves via the oxPL/TLR4 axis — status “amplifies inflammation”. Ferroptosis of activated hepatic stellate cells reduces fibrogenesis — status “anti-fibrotic”. Adaptive and innate lymphoid cells: altered T- and B-cell homeostasis together with ferroptosis of dendritic and natural killer cells lowers immune surveillance during progression from MASH to hepatocellular carcinoma — status “context-dependent”. Liver sinusoidal endothelial cells, which form the vascular interface: ferroptosis and calcium-driven defenestration increase the delivery of oxidisable lipid to hepatocytes and remove the tonic restraint on stellate cell activation; the status is given as “damaging (plausible)” because the direct evidence derives mainly from cold-storage and ischaemia–reperfusion models rather than from dietary MASLD models. The symbol ⇄ in the tier heading denotes the self-sustaining hepatocyte ⇄ macrophage loop. The bar at the base of the tier indicates the net tissue trajectory: steatosis → MASH → fibrosis → cirrhosis/HCC. The vertical arrow to tier 4 is labelled “therapeutic implications”. Tier 4 — Clinical translation. Patient stratification by a ferroptosis signature (↓GPX4, ↑4-HNE, redox-active Fe²^+^) is proposed as the entry criterion for biomarker-led randomised trials rather than all-comer designs. Module-matched therapies are listed in the order of the modules in tier 2 (iron chelation; ACSL4 and lipid-remodelling inhibitors; radical scavengers and ferroptosis inhibitors; NRF2/HO-1 activators and parallel defence circuits). As stated on the image, all of them remain preclinical and none is a selective ferroptosis modulator. Non-invasive read-outs (protein biomarkers, circulating miRNAs) together with cell-specific delivery close the loop back to tier 3. The image was created by the authors in Microsoft PowerPoint (Microsoft Corporation, Redmond, WA, USA).

Thus, in the classical model, three inextricably linked components are required for the initiation of ferroptosis. The first component is redox-active iron, capable of catalyzing the formation of reactive oxygen species and accelerating lipid peroxidation chain reactions. The second component is membrane PUFA phospholipids, which serve as a substrate for peroxidation. The third component is a deficiency in antioxidant defense, primarily in the Xc^-^/GSH/GPX4 axis (54–59). These components of ferroptosis are closely linked to the liver’s fundamental metabolic functions, making this organ particularly vulnerable to this type of cell death. The liver serves as the body’s primary iron store and, at the same time, as the central organ of lipid metabolism, which includes the synthesis, oxidation, and export of lipids (27, 60, 61). This functional coupling increases the risk of uncontrolled interactions between redox-active iron, easily oxidizable polyunsaturated fatty acids (PUFAs) in membrane phospholipids, and the pro-inflammatory/oxidative stress environment characteristic of MASLD and MASH. Taken together, these metabolic features allow ferroptosis to be considered one of the key mechanisms of hepatocyte damage in MASLD/MASH, especially in patients with signs of iron metabolism disorders, increased lipid peroxidation, and impaired GPX4/GSH-dependent protection (27, 60, 61).

The link between MASLD and ferroptosis is generally viewed as a pathological dysregulation of three closely interrelated metabolic axes. The first axis regulates iron metabolism and includes its uptake into hepatocytes via TFRC/TFR1, DMT1/SLC11A2, and SLC39A14, iron storage in ferritin, export via ferroportin/SLC40A1, and iron release during NCOA4-dependent ferritinophagy. The second axis covers lipid metabolism. Polyunsaturated fatty acids (PUFAs) are incorporated into membrane phospholipids with the participation of long-chain acyl-coenzyme A (CoA) synthase 4 (ACSL4) and LPCAT3, after which they undergo oxidation via both enzymatic (lipoxygenases, POR) and non-enzymatic (Fenton reaction) pathways. The third axis is represented by antioxidant defense, the central component of which is the GPX4/GSH system. Parallel protective mechanisms include FSP1/Coenzyme Q10 (CoQ10), GCH1/5,6,7,8-Tetrahydrobiopterin (BH4), and nuclear factor erythroid 2-related factor 2 (NRF2, encoded by *NFE2L2*)-dependent protective programs (54, 57, 62). In MASH, these metabolic axes shift toward increased vulnerability to ferroptosis, with corresponding pro-ferroptotic changes already detected at the stage of isolated steatosis. Thus, the concept of “oxidative damage” is given concrete molecular substance in the form of a specific type of regulated cell death that plays a key role in disease progression (28).

Data obtained from humans directly confirm heterogeneity of ferroptosis. A subgroup with a ferroptosis signature was identified in liver biopsies from patients, characterized by signs of cell death, accumulation of 4-hydroxynonenal (4-HNE), the presence of redox-active Fe²^+^, and a reduction in GPX4-dependent protection (30). However, these data do not imply that ferroptosis dominates in all patients with MASLD/MASH. In some patients, alternative modes of regulated cell death (e.g., apoptosis, necroptosis) dominate the pathogenesis and/or effective anti-ferroptotic adaptations are preserved (e.g., through the expression of ferroptosis suppressor protein 1 (FSP1) or GTP cyclohydrolase 1 (GCH1)) (30).

## 4 Molecular mechanisms of ferroptosis in the liver in MASLD

### 4.1 Iron module of ferroptosis

Iron plays a role in ferroptosis not only as a background factor in oxidative stress but also as a dynamic regulator of the rate of membrane peroxidation. Under conditions of iron overload, including that resulting from ferritinophagy, the labile iron pool (LIP) of ferrous iron (Fe²^+^) increases. This pool serves as a direct catalyst for the Fenton reaction, accelerating the formation of highly reactive hydroxyl radicals and, consequently, lipid peroxides (63, 64). An increase in intracellular LIP may result from several interrelated processes, such as dysregulation of systemic iron metabolism—particularly the hepcidin–ferroportin axis—and changes in the expression of key iron importers, such as transferrin, transferrin receptor 1 (TFR1)/*TFRC*, DMT1/*SLC11A2*, and ZIP14/*SLC39A14*. Additionally, enhanced iron release from intracellular stores, mediated by NCOA4-dependent ferritinophagy (63, 64). These molecular mechanisms are reflected in clinical practice. A clinically significant observation is the high frequency of stainable hepatic iron in MASH, reaching 41% in large cohorts (65, 66). The pattern of iron deposition is of fundamental importance: in the largest biopsy cohort of the nonalcoholic steatohepatitis (NASH) Clinical Research Network (n=2833), the presence of iron in cells of the reticuloendothelial system (RES), predominantly in Kupffer cells, was independently associated with more severe lobular inflammation, marked ballooning of hepatocytes, and an increased likelihood of advanced fibrosis (adjusted OR: 1.34, 95% CI: 1.11–1.62, p=0.003) compared with patients without iron or with isolated hepatocellular (HC) iron (66). These data are consistent with a model in which iron accumulation causes oxidative damage, leading to ferroptosis and inflammation. Support for this model comes from the detection of elevated serum levels of malondialdehyde (MDA) in patients with RES-iron, as well as fragments of cytokeratin-18 (CK-18), reflecting the intensity of apoptosis (67). It should be noted that MDA, 4-HNE, CK-18, and tissue iron are markers of oxidative damage, cell death, or iron metabolism disorders, but are not strictly specific to ferroptosis. Therefore, a diagnosis of ferroptosis requires a combination of several criteria: labile Fe²^+^, decreased GPX4/GSH, accumulation of oxidized PUFA phospholipids, and reversibility of damage upon the use of ferroptosis inhibitors or iron chelators.

The molecular basis for the described clinical associations lies in multiple dysregulations in the expression of genes involved in iron metabolism and ferroptosis, which create a pro-ferroptotic background. Liver transcriptome studies confirm this finding, demonstrating that disease progression is accompanied by profound dysregulation of genes controlling intracellular iron homeostasis. In particular, patients with MASLD/MASH exhibit increased expression of the main iron importers—the TFR1 and the divalent metal transporter DMT1/SLC11A2—while the mRNA level of the sole iron exporter, ferroportin FPN1/*SLC40A1*, is, conversely, reduced (27, 29). The involvement of another importer—SLC39A14, which mediates the uptake of non-transferrin-bound iron by hepatocytes—in the pathogenesis is confirmed by the fact that its genetic deletion suppresses ferroptosis and limits the development of liver fibrosis (27). Concurrent with increased import, intracellular iron stores are mobilized. NCOA4-dependent ferritinophagy is activated, ensuring lysosomal degradation of ferritin and the release of free Fe²^+^, which further expands the labile iron pool (27). These changes indicate a systemic shift in the balance of iron transport toward its accumulation in hepatocytes.

Among the ferroptotic modules, there is increased expression of *ACSL4*—a key enzyme that facilitates the incorporation of polyunsaturated fatty acids into membrane phospholipids and thereby provides a substrate for peroxidation (28, 29). Concurrently, a decrease in the expression of the central antioxidant enzyme *GPX4* is observed, indicating a weakening of cellular protection against the accumulation of lipid hydroperoxides and contributing to the onset of ferroptotic hepatocyte death (28, 29).

Thus, the convergence of three processes—increased iron influx, suppression of its export, and activation of ferritinophagy—combined with an imbalance of pro- and antioxidant enzymes, creates a sustained pro-ferroptotic background. This pro-ferroptotic background increases the likelihood of ferroptotic cell death under additional metabolic or inflammatory stress. The key event triggering peroxidation is the accumulation in membranes of a specific class of lipids that serve as substrates for radical reactions. The mechanisms of their formation are described below.

### 4.2 Lipid module of ferroptosis

Ferroptosis requires the presence of a vulnerable substrate—phospholipids containing polyunsaturated fatty acids (PUFA phospholipids). The classical pathway of ferroptosis-dependent lipid remodeling involves the sequential activation of enzymes that incorporate PUFAs into phospholipid tails. ACSL4 catalyzes the formation of PUFA thioesters with coenzyme A (PUFA-CoA), while lysophosphatidylcholine acyltransferase 3 (LPCAT3) transfers the activated PUFA to the sn-2 position of phospholipids, primarily phosphatidylethanolamine (PE). Collectively, these reactions lead to the enrichment of membranes with easily oxidizable PUFA-PE, thereby creating the substrate basis for ferroptosis (68–70).

The formation of lipid hydroperoxides from PUFA-PE-enriched membranes occurs via two pathways. Within the enzymatic mechanism, iron-containing lipoxygenases, primarily ALOX15 (15-lipoxygenase-1), in complex with the chaperone protein phosphatidylethanolamine-binding protein 1 (PEBP1), directly oxidize PUFA-PE to form 15-hydroperoxy-arachidonoyl-phosphatidylethanolamine (15-HpETE-PE)—a key lipid death signal. The non-enzymatic pathway is mediated by free-radical chain reactions initiated by the Fenton reaction, during which lipid hydroperoxides (PUFA-PLOOH) are also generated from PUFA phospholipids. Both pathways lead to the accumulation of PUFA-PLOOH, which serve as direct death signals and effectors of ferroptosis (71, 72). Thus, the execution of ferroptosis critically depends on the presence in membranes of the primary substrate for peroxidation—phospholipids containing polyunsaturated fatty acids, primarily PUFA-PE. A critical step in PUFA-PE biosynthesis is the activation of free polyunsaturated fatty acids, a process mediated by the family of long-chain fatty acid acyl-CoA synthetases. The ACSL4 isoform (long-chain fatty acid CoA ligase 4) plays a key role in the context of ferroptosis. ACSL4 catalyzes the conversion of free polyunsaturated fatty acids, primarily arachidonic and adrenic acids, into their thioesters with coenzyme A (CoA) (arachidonoyl-CoA (AA-CoA) and adrenic acid-CoA (AdA-CoA)) (71, 73, 74). The formation of these PUFA-CoAs is a necessary prerequisite for the subsequent enzymatic incorporation of arachidonic and adrenic acids into the sn-2 position of phospholipids, primarily phosphatidylethanolamine (PE). It is precisely these molecules—PE-AA and PE-AdA—that, upon oxidation, form a membrane environment vulnerable to peroxidation, the direct substrate of ferroptosis (71, 74).

In the context of MASLD, the role of ACSL4 has been confirmed by several independent lines of evidence. First, in patients with MASLD/MASH, the levels of ACSL4 mRNA and protein in the liver are significantly higher than in healthy individuals, and the expression level of *ACSL4* positively correlates with the degree of steatosis and liver fat content (75, 76). Elevated ACSL4 expression has been documented in three independent transcriptomic cohorts (GSE66676, GSE63067, GSE48452) and confirmed immunohistochemically (75, 76). Second, hepatocyte-specific inactivation of ACSL4 (HepKO) in mice, according to some researchers, protects against the development of steatosis, inflammation, and fibrosis, accompanied by a decrease in transaminases and improved histology. The study was conducted using several dietary models, including a high-fat diet (high-fat, cholesterol, and fructose diet (HFD)), a methionine-choline-deficient diet (MCD), and a diet high in fat, cholesterol, and fructose (HFF) (76). However, in a recent study using other diets (Choline-deficient high-fat diets (CD-HFD) and Western diet), a similar ACSL4 knockout did not exert a protective effect on steatosis, inflammation, or fibrosis, suggesting that the effect may depend on the type of diet and housing conditions (77). Third, abemaciclib, known as a cyclin-dependent kinase 4/6 (CDK4/6) inhibitor, demonstrated the ability under experimental conditions to reduce ACSL4-associated ferroptotic vulnerability and improve the NASH phenotype in mice by reducing steatosis, inflammation, and fibrosis in models fed high-fat and methionine-choline-deficient diets (75, 76). However, its effects in NASH cannot be unequivocally attributed to direct inhibition of ACSL4, as the drug exhibits pronounced CDK4/6-dependent and potentially other off-target effects. Therefore, abemaciclib should be considered not as a specific ACSL4 inhibitor in MASLD/MASH, but as an experimental pharmacological tool highlighting the potential significance of ACSL4-associated ferroptotic vulnerability.

Taken together, these findings position ACSL4 as one of the most well-established ferroptosis-dependent mediators of MASH and a promising therapeutic target, particularly for a molecularly stratified subgroup of patients with proven PUFA-phospholipid and ferroptosis vulnerability.

For the lipid module of ferroptosis, genes that control the pool of available polyunsaturated fatty acids (PUFAs) and their incorporation into phospholipids are of critical importance. These primarily include enzymes involved in the biosynthesis and elongation of ω-6 PUFAs: the desaturases FADS1 and FADS2, as well as the elongase ELOVL5 (together with ELOVL2), which catalyze the conversion of linoleic acid into arachidonic (AA) and adrenic (AdA) acids (78–80). In addition, genes controlling phospholipid remodeling enzymes. Central among these is lysophosphatidylcholine acyltransferase 3 (LPCAT3), which primarily incorporates PUFAs into phosphatidylcholine and phosphatidylethanolamine (78, 80, 81).

It has been experimentally demonstrated that the activity of the desaturation (FADS1/2) and elongation (ELOVL5) pathways, which mediate the synthesis of arachidonic and adrenic acids from linoleic acid, directly influence cellular sensitivity to ferroptosis by modulating the content of PUFA-phospholipids in membranes (78, 79). In the context of MASLD/MASH, reduced activity of the desaturase FADS1 (D5D) and alterations in the function of the elongase ELOVL5 are associated with the progression of MASLD/MASH, which is accompanied by a decrease in the content of long-chain PUFAs in the liver (82–84). Thus, ferroptosis sensitivity is determined not by the absolute content of all long-chain PUFAs in the liver, but by their distribution among membrane phospholipids, the degree of ACSL4/LPCAT3-dependent incorporation into PE, the PUFA/monounsaturated fatty acids (MUFA) balance, and the efficiency of GPX4-dependent detoxification of lipid hydroperoxides.

The role of LPCAT3 also requires contextual interpretation. On the one hand, LPCAT3 promotes the incorporation of PUFAs into phospholipids and thereby increases the pool of lipids vulnerable to peroxidation. On the other hand, LPCAT3 is essential for normal membrane composition, lipoprotein metabolism, and mitochondrial function in the liver (29, 85, 86). Therefore, a decrease or increase in LPCAT3 activity should not automatically be interpreted as an unambiguously protective or damaging event. The net effect depends on the balance between the availability of PUFA phospholipids, GPX4/GSH activity, labile iron, and the stage of the disease.

In addition to classical diacyl PUFA phospholipids, ferroptosis sensitivity can be modulated by peroxisome-dependent ether lipids. This pathway includes the rate-limiting enzyme fatty acyl-CoA reductase 1 (FAR1) (1-hexadecanol and 1-octadecanol derived from palmitic (C16:0) and stearic (C18:0) fatty acids), glyceronephosphate O-acyltransferase (GNPAT) (the first step in ether backbone assembly), alkylglycerone phosphate synthase (AGPS) (formation of an O-alkyl bond at the sn-1 position of the glycerophospholipid), and enzymes involved in plasmalogen synthesis (87–89).

Peroxisomes are capable of enhancing susceptibility to ferroptosis by facilitating the synthesis of alkyl-ester polyunsaturated phospholipids (PUFA-ester phospholipids). These lipids serve as an additional substrate for iron-dependent peroxidation. Their production requires a functional peroxisomal biogenetic machinery, including proteins of the peroxisomal biogenesis factor 1 (PEX) family (in particular, PEX3, PEX10, PEX12, PEX16, and PEX19, the loss of which reduces sensitivity to ferroptosis) (88). In contrast, plasmalogens (vinyl-ester PUFA lipids), generated from alkyl-ester precursors by the transmembrane protein 189 (TMEM189) enzyme, may limit ferroptosis, including by suppressing the expression of FAR1 and, consequently, the formation of its pro-ferroptotic precursors (87–89).

Thus, components of the peroxisomal-ester lipid pathway are directly involved in the regulation of ferroptosis. However, their role in the pathogenesis of MASLD/MASH has been characterized inconsistently. According to data obtained from tumor cell lines and a renal ischemia model, FAR1 is a key enzyme for the synthesis of alkyl-ether PUFA phospholipids, which serve as substrates for lipid peroxidation and enhance ferroptosis (87). Given the central role of ferroptosis in the pathogenesis of MASLD/MASH, it can be assumed that FAR1 contributes to disease progression. However, targeted studies confirming this hypothesis in models of steatosis/steatohepatitis are currently lacking. The role of GNPAT in the progression of MASLD/MASH is due to its function as the rate-limiting enzyme in plasmalogen biosynthesis: the decrease in *Gnpat* expression observed in steatohepatitis leads to a drop in the levels of docosahexaenoic acid (DHA)-containing plasmalogens, suppression of peroxisome proliferator activated receptor-alpha (PPARα)-dependent mitochondrial and peroxisomal fatty acid oxidation, and increased sensitivity of hepatocytes to steatosis and inflammation (90). Direct evidence of AGPS involvement in the progression of MASLD/MASH is currently limited (91). It is hypothesized that the potential contribution of this enzyme may occur through impaired plasmalogen synthesis and altered oxylipin balance. However, this hypothesis requires experimental validation in models of steatosis and steatohepatitis. Collectively, disturbances in peroxisomal homeostasis, including defects in the synthesis of ether lipids, contribute to the accumulation of pro-ferroptotic PUFA-phospholipids and the weakening of hepatocyte antioxidant defense, which exacerbates oxidative damage and inflammation in MASLD (91). Thus, enzymes of the peroxisomal-ester lipid pathway can be considered potential targets for therapeutic intervention, although their specific role in disease progression requires further study and validation in cellular models of MASLD and MASH, microphysiological systems (MPS) systems, and animal dietary models.

### 4.3 Distal cholesterol biosynthesis and 7-dehydrocholesterol: an endogenous radical-trapping antioxidant

In addition to the ACSL4/LPCAT3 axis and the peroxisomal pathway for the synthesis of esterified lipids, membrane sensitivity to peroxidation is also determined by the final stage of cholesterol biosynthesis. In two independent studies published simultaneously in Nature in 2024, 7-dehydrocholesterol (7-DHC)—the direct substrate of 7-dehydrocholesterol reductase (DHCR7) at the terminal stage of the Kandutsch-Russell pathway—was identified as an endogenous membrane-associated suppressor of ferroptosis (92, 93). Unlike cholesterol, 7-DHC contains a conjugated 5,7-diene system, which accounts for its exceptionally high reactivity toward lipid peroxyl radicals. By incorporating into the lipid bilayer, 7-DHC functions as a radical-scavenging sterol and competes with polyunsaturated phospholipids for interaction with peroxyl radicals, taking on the burden of radical chain oxidation, thereby limiting the spread of chain peroxidation and damage to plasma and mitochondrial membranes (92, 93). Genetic or pharmacological inhibition of DHCR7 is accompanied by the accumulation of its substrate 7-DHC and increases cellular resistance to ferroptosis (92, 93). In contrast, genetic inactivation of the enzymes CYP51A1, MSMO1, EBP, and SC5D, which are located further upstream in the distal cholesterol biosynthesis pathway, reduces intracellular 7-DHC levels and increases sensitivity to GPX4 inhibition (92). These results indicate that 7-DHC levels are one of the key factors through which enzymes in the distal cholesterol biosynthesis pathway modulate ferroptosis sensitivity. Consequently, DHCR7 may act as a context-dependent positive regulator of ferroptosis susceptibility, as it catalyzes the conversion of the endogenous anti-ferroptotic metabolite 7-DHC into cholesterol. This mechanism was independently confirmed by Yamada et al. in Huh-7 cells and primary hepatocytes (94). However, the anti-ferroptotic effect of 7-DHC should not be interpreted as unconditionally cytoprotective. Its free-radical oxidation is accompanied by the formation of reactive oxysterols, whose potential toxicity must be considered in light of the cell type, the concentration of 7-DHC, and the intensity of oxidative stress.

Functionally, this places 7-DHC in the same class as the radical scavengers discussed in the section on therapeutic prospects, but with two fundamental differences. First, unlike α-tocopherol, ferrostatin-1, and liproxstatin-1, 7-DHC is not an exogenous agent but a constitutive intermediate in sterol metabolism. Therefore, its concentration is determined by the flux through the mevalonate and distal cholesterol pathways, rather than by the dosing regimen. Second, its protective effect is exerted within the bilayer, directly at the site of chain extension, which offers a possible explanation for why cells with similar GPX4 activity can differ markedly in their ferroptosis threshold. In this sense, the 7-DHC axis complements, rather than duplicates, the classical Xc^-^/GSH/GPX4 and FSP1/CoQ10 pathways. Since MASLD/MASH is characterized by profound alterations in cholesterol metabolism, including the accumulation of free cholesterol in hepatocytes and changes in the expression of distal biosynthetic enzymes, 7-DHC-dependent modulation of the ferroptosis threshold in a steatotic liver is biologically plausible. However, it should be noted that this remains a hypothesis, as no study has measured hepatic 7-DHC levels or tested the effects on DHCR7 in a dietary MASH model. Consequently, the relevance of this pathway for MASLD and MASH requires validation in hepatocytes, macrophages, and *in vivo* models. A separate caveat concerns the evidence base itself. The journal Nature published an Editor’s Note regarding one of the two seminal articles (92), indicating that doubts had been raised about the reliability of the data presented therein. This work should be cited with appropriate caution, and the main support for the concept should be considered to be the independent replication of the key observation in a parallel article (93) and in a study on hepatocytes (94).

### 4.4 Oxidative module of ferroptosis

The peroxidation of polyunsaturated fatty acids (PUFAs) within membrane phospholipids during ferroptosis occurs via two main pathways. The first is enzymatic. It is mediated by iron-containing lipoxygenases. In humans, six isoforms of these enzymes are expressed: ALOX5, ALOX12, ALOX12B, ALOX15, ALOX15B, and ALOXE3. Lipoxygenases catalyze the stereospecific dioxygenation of PUFAs (95). The second pathway is non-enzymatic. It involves free-radical chain auto-oxidation. This reaction is accelerated in the presence of labile iron via the Fenton reaction (78, 95). Lipoxygenases can act as initiators of lipid hydroperoxide formation, especially under conditions of glutathione depletion, whereas in the case of direct GPX4 inhibition, non-enzymatic propagation of peroxidation plays a dominant role (95). The key factors determining the rate and nature of peroxidation propagation are the availability of redox-active iron and the molecular architecture of the membrane—in particular, the ratio of PUFAs (especially arachidonic and adrenic acids) to monounsaturated fatty acids. In addition, the process is influenced by the activity of phospholipid remodeling enzymes. Key among these are ACSL4 and LPCAT3 (78). However, the effectiveness of any of these peroxidation mechanisms is ultimately limited by the amount of available redox-active iron. An important, though less studied, link connecting the lipid substrate to iron-dependent oxidation is the enzyme PHKG2 (phosphorylase kinase, catalytic subunit G2). In fundamental screening studies using shRNA screening (95), it was shown that PHKG2 regulates the level of the labile iron pool in cells. PHKG2 knockdown reduced intracellular iron, prevented the accumulation of lipid peroxides, and suppressed ferroptosis induced by the Xc^-^ system inhibitor (erastin). Although PHKG2 is classically associated with the activation of glycogen phosphorylase during glycogenolysis, its action in the context of ferroptosis is not mediated by glycogenolysis (glycogen phosphorylase inhibitors did not affect ferroptosis). Consequently, PHKG2 possesses a previously unknown function that modulates iron-dependent lipid peroxidation (95). This function of PHKG2 may be directly relevant to the pathogenesis of MASLD/MASH, where iron accumulation in the liver and hepatocyte ferroptosis are considered important factors in disease progression. It can be hypothesized that increased PHKG2 activity in hepatocytes contributes to the expansion of the labile iron pool, thereby enhancing the peroxidation of membrane phospholipids and increasing cellular susceptibility to ferroptosis. Conversely, a decrease in PHKG2 activity or expression could limit iron availability for Fenton and lipoxygenase reactions, reducing the severity of lipid peroxidation and hepatocyte damage. However, there is currently insufficient direct evidence of its involvement in the development of steatosis or MASH. Therefore, it is appropriate to refer to PHKG2 not as an established pathogenic factor of MASLD, but as a potential regulator linking energy metabolism, iron-dependent peroxidation, and ferroptosis sensitivity.

In contrast, the role of the classical axis linking GPX4, lipoxygenases, and iron availability has been directly confirmed in steatohepatitis models. In *in vivo* experiments in mice with NASH induced by a methionine-choline-deficient (MCD) diet, a key role for GPX4-dependent ferroptosis was demonstrated. Pharmacological inhibition of GPX4 using RSL3 led to a decrease in GPX4 protein levels in the liver, an increase in 12/15-lipoxygenase (12/15-LOX) activity, and the accumulation of lipid peroxidation markers (malondialdehyde, MDA), and depletion of GSH. This was accompanied by increased steatosis, inflammation, and hepatocyte death (apoptosis-inducing factor, AIF), i.e., it exacerbated the severity of NASH (96). In contrast, activation of GPX4 using sodium selenite, chelation of labile iron with deferoxamine (DFO), and direct inhibition of ferroptosis with liproxastatin-1 (Lip-1) reduced levels of 12/15-LOX and MDA, restored GSH, and reduced steatosis, inflammation, and hepatocyte death, thereby mitigating the course of NASH. These data suggest that an imbalance in the GPX4-12/15-LOX-iron axis is a critical mechanism in the progression of steatohepatitis, and that modulation of ferroptosis is a promising therapeutic target (96).

In addition to lipoxygenases, NADPH oxidases (97–99), electron transfer enzymes (100–102), and regulatory proteins (103) contribute to the generation of reactive oxygen species (ROS) and the initiation of lipid peroxidation during ferroptosis. NADPH oxidase 1 and 2 (NOX1 and NOX2) primarily generate superoxide anion (O_2_^-^), whereas NOX4, due to the structural features of its E-loop, directly produces hydrogen peroxide (H_2_O_2_) (97–99). Electron transfer enzymes, such as NADPH-cytochrome P450 reductase (POR) and NADH-cytochrome b5 reductase 1 (CYB5R1), catalyze the formation of H_2_O_2_, which, in the presence of iron(II), generates hydroxyl radicals via the Fenton reaction, initiating the peroxidation of polyunsaturated fatty acids (100–102). The activity of NADPH oxidases in ferroptosis may be positively regulated by regulatory proteins. For example, dipeptidyl peptidase 4 (DPP4, also known as CD26), by binding to NOX1, enhances ROS production and may be associated with ferroptosis (103). However, the involvement of DPP4 in ferroptosis specifically in MASLD/MASH should currently be considered biologically plausible but not yet sufficiently clinically validated.

NOX systems in MASLD have cell-specific significance. ROS generation involving NADPH oxidases takes on particular pathogenic significance, as these enzymes not only provide substrates for peroxidation reactions but are also directly involved in key stages of disease progression. The involvement of NOX1 and NOX2 in the progression of MASLD/MASH is mediated by their ability to generate reactive oxygen species, which activate Kupffer cells, enhance the production of pro-inflammatory cytokines (TNFα, IL-6, IL-1β), and promote the transdifferentiation of stellate cells into profibrogenic myofibroblasts (104–106). NOX4 exhibits a dual role. In hepatocytes, it may support *NFE2L2*/NRF2-dependent antioxidant adaptation and limit the progression of steatosis in NASH, whereas in hepatic stellate cells (HSCs), NOX4 promotes transforming growth factor (TGF)-β (TGF-β)-dependent fibrogenic activation (104–107). Consequently, NOX4 cannot be viewed solely as a deleterious ROS generator. Its systemic inhibition may have unpredictable consequences and requires cell-specific evaluation.

Along with the listed enzymatic sources, mitochondrial dysfunction makes a significant contribution to ROS generation in MASLD. The accumulation of free fatty acids and increased β-oxidation enhance electron flow in the respiratory chain, leading to electron leakage and the one-electron reduction of oxygen to form the superoxide anion (O_2_^-^) (106). Mitochondrial O_2_^-^ rapidly dismutates to H_2_O_2_, which, in the presence of labile iron, participates in the Fenton reaction, generating hydroxyl radicals. Furthermore, mitochondrial ROS can induce NOX4 expression via NFE2L2 activation, thereby closing the vicious cycle of oxidative stress in hepatocytes (107). Mitochondria not only serve as a direct source of pro-oxidants but also potentiate other peroxidation mechanisms. Collectively, in MASLD, a unique situation arises in which multiple enzymatic and non-enzymatic sources of ROS (lipoxygenases, NADPH oxidases, POR/CYB5R1, the mitochondrial respiratory chain) operate under conditions of increased availability of redox-active iron, creating ideal conditions for avalanche-like lipid peroxidation and ferroptosis. Whether this cascade culminates in ferroptotic death depends on the capacity of the antioxidant circuits.

### 4.5 Mitochondrial dysfunction under lipotoxic and hyperglycemic stress

Mitochondria occupy a special position in this scheme, as they are simultaneously the largest source of reactive oxygen species in hepatocytes, the primary site of fatty acid utilization, and the organelle whose failure converts metabolic overload into a death signal. In the early stages of MASLD, the liver responds to excess substrate with mitochondrial adaptation—increased β-oxidation, enhanced respiratory capacity, and biogenesis (108). However, this adaptation is not sustainable. As lipid influx continues to exceed the oxidative capacity of the mitochondria, incomplete β-oxidation leads to the accumulation of acylcarnitine intermediates, and the electron flow through complexes I and III becomes increasingly decoupled from ATP synthesis. As a result, electron leakage increases, which enhances the one-electron reduction of oxygen to superoxide (108, 109). These changes create a metabolic backdrop for oxidative stress and subsequent damage to hepatocytes. Hyperglycemia affects the same targets in a different way. Excess glucose flow through glycolysis and the tricarboxylic acid cycle increases the formation of the reduced cofactors NADH and FADH_2_, which is accompanied by an increase in the NADH/NAD^+^ ratio and an enhanced flow of electrons into the mitochondrial respiratory chain (110–112). When the cell’s demand for ATP is relatively low, a high proton electrochemical gradient forms and hyperpolarization of the inner mitochondrial membrane develops, which slows electron transport along the respiratory chain and increases the lifetime of reduced intermediate carriers, primarily ubisemiquinone, thereby increasing the likelihood of electron leakage to molecular oxygen with the formation of superoxide anion, primarily at complexes I and III (113–115). Excessive production of mitochondrial superoxide is considered one of the key early events that initiate the activation of the polyol pathway, the formation of AGEs, the activation of hepatocytes activate protein kinase C (PKC), and the hexosamine pathway during hyperglycemia (110, 116). In liver cells, prolonged hyperglycemia is accompanied by impaired mitochondrial bioenergetics (37, 117). Lipotoxic stress, caused by an excess of free fatty acids, also leads to mitochondrial dysfunction (118). When hyperglycemia and lipotoxicity act in combination, these effects are additive, indicating a convergence of the glucotoxic and lipotoxic cascades at the mitochondrial level. The result is a shift in mitochondrial quality control: the expression of fission proteins dynamin-related protein 1 (Drp1) and protein Fission 1 homologue (Fis1) increases, while the levels of fusion proteins mitofusins 1 and 2 (Mfn1/2) and optic atrophy 1 (Opa1) decrease, so that fission predominates over fusion (119–121). At the same time, PINK1/Parkin-dependent mitophagy is suppressed, resulting in insufficient elimination of damaged organelles, and a pool of dysfunctional mitochondria persists in the cell as a chronic pro-oxidant source (119–121).

Three characteristics of this state are crucial for ferroptosis. Mitochondrial superoxide dismutates to hydrogen peroxide (122), which, in the presence of an expanded labile pool of ferrous iron, fuels the Fenton reaction and initiates the chain peroxidation of PUFA phospholipids (123). Mitochondria themselves serve as a compartment for iron transport and the assembly of iron-sulfur clusters (124, 125). Therefore, their dysfunction—such as a decrease in frataxin expression—releases, rather than binds, catalytically active iron. Finally, mitochondrial reactive oxygen species induce NOX4 expression via NFE2L2, closing a positive feedback loop with non-mitochondrial oxidases (107). Accordingly, mitochondrial damage in MASLD should not be interpreted merely as a marker of metabolic stress. In other words, mitochondrial damage increases hepatocyte susceptibility to ferroptosis death.

### 4.6 Disruption of calcium homeostasis as a consequence of lipid peroxidation

Lipid peroxidation damages membranes not only structurally. The electrophilic aldehydes formed during this process, such as 4-hydroxynonenal, directly modify the Sarcoendoplasmic Reticulum Calcium ATPase (SERCA) calcium pump, inhibiting its activity and disrupting calcium homeostasis in the endoplasmic reticulum (126). Furthermore, the accumulation of lipid intermediates in hepatocytes activates protein kinase C delta (PKCδ), which also inhibits SERCA, contributing to endoplasmic reticulum stress (127). Aldehyde products of peroxidation, primarily 4-hydroxynonenal and malondialdehyde, are capable of forming adducts with cysteine, histidine, and lysine residues in proteins (128), including, potentially, transport ATPases and ion channels. At the same time, the accumulation of hydroperoxides in membranes leads to a loss of order in the lipid bilayer, activation of mechanosensitive cation channels, and inhibition of Na^+^/K^+^-ATPase activity, which collectively sharply increases nonspecific cation permeability (129–131). A logical consequence is an increase in the concentration of free cytosolic calcium, which begins even before the plasma membrane’s integrity is clearly compromised.

Direct evidence for this sequence of events in ferroptosis has already been obtained. In cells dying via the ferroptosis pathway, nanometer-scale pores form in the plasma membrane, allowing Ca²^+^ to enter long before the membrane ruptures. Calcium influx is oscillatory rather than monotonic and recruits ESCRT-III-dependent membrane repair, which, in turn, determines the kinetics of cell death (132). Thus, calcium is not a passive marker of the terminal stage of cell death, but rather a regulatable factor that determines whether a cell with oxidized membranes will die or recover. Accordingly, calcium signals modulate ferroptosis sensitivity in both directions depending on the compartment involved, the ion source, and the cell’s buffering capacity (133). In this context, the lysosomal calcium-permeable channels two-pore channel 2 (TPC2) and mucolipin 1 (TRPML1) alter susceptibility to ferroptosis in hepatocyte-derived cells (134). The terminal stage—namely, plasma membrane rupture with the release of danger signals—is mediated by the ninjurin-1 (NINJ1) protein, which links the physical endpoint of ferroptosis to its immunogenicity (135).

In a steatotic liver, this cascade occurs against the backdrop of an already impaired calcium system. MASLD is accompanied by depletion of the calcium stores in the endoplasmic reticulum, impaired function of Ca²^+^-ATPases in the sarcoplasmic and endoplasmic reticulum, and remodeling of mitochondria-associated membranes, resulting in dysregulation of Ca²^+^ transport into mitochondria via inositol-1,4,5-trisphosphate receptors. This organelle-level collapse of calcium homeostasis has been proposed as an integrative mechanism underlying the progression of MASLD, which also alters hepatic immune signaling (136). Mitochondrial calcium overload represents a functionally validated node in this network. Inhibition of the hepatic mitochondrial calcium uniporter (MCU) reduced the severity of steatohepatitis and fibrosis in mice (137). In a model of chemically induced steatohepatitis, an autophagy-dependent increase in MCU caused mitochondrial calcium overload and stimulated ferroptosis through direct interaction of MCU with ACSL4 (138). Cytosolic calcium overload also has a cell-specific vascular equivalent. It causes defenestration of hepatic sinusoidal endothelial cells, and its pharmacological inhibition reduces the severity of experimental steatotic liver disease (139). Mechanosensitive Ca²^+^-permeable channels of the Piezo family, primarily Piezo1, provide an additional pathway through which the increasing stiffness of the fibrotic liver is converted into intracellular calcium signals (140). Thus, calcium plays a dual role in the process of ferroptosis. It is a consequence of oxidative damage to membranes. At the same time, it helps determine whether this damage will be lethal. This effect is mediated through mitochondrial calcium overload, the involvement of ACSL4, and the activation of membrane repair pathways. Quantitative measurement of calcium dynamics is not part of the standard set of criteria for ferroptosis, and its inclusion in MASLD studies represents one of the most accessible ways to verify whether the peroxidation observed in the tissue has functional consequences.

### 4.7 Antioxidant protection against ferroptosis: from molecular mechanisms to dysregulation in MASLD/MASH

The Xc^-^ system serves as the central component of classical protection against ferroptosis. This heterodimer is formed by the SLC7A11 and SLC3A2 subunits. The Xc^-^ system mediates the exchange of extracellular cystine for intracellular glutamate (141). Once inside the cell, cystine is reduced to cysteine. This reaction requires NADPH, which serves as an electron donor (141). Cysteine is the limiting substrate for the synthesis of GSH — a tripeptide consisting of glutamate, cysteine, and glycine residues (141). GSH acts as a cofactor for GPX4 — a selenoprotein capable of converting toxic lipid hydroperoxides into non-toxic lipid alcohols. In this way, GPX4 prevents chain peroxidation of membranes (141). NADPH is also consumed for the regeneration of GSH from its oxidized form, GSSG (141).

SLC7A11 expression is subject to multilevel transcriptional control. Activating signals enhance gene transcription. These include activating transcription factor 4 (ATF4), which is induced by amino acid starvation, and NRF2, which is activated under oxidative stress conditions. Repressive signals, on the other hand, suppress SLC7A11 expression. An example is the tumor suppressor TP53/p53. These signals are integrated through the Kelch-like ECH-associated protein 1 (KEAP1)–NRF2 regulatory loop, which ultimately determines the cell’s sensitivity threshold to ferroptosis (142).

It is precisely the imbalance of this system that underlies the vulnerability to ferroptosis in a significant proportion of patients with MASLD. Using liver biopsy samples from patients with MASLD, machine learning methods identified a subgroup (approximately 40–60% of cases) characterized by reduced levels of the key ferroptosis protector GPX4, accumulation of 4-hydroxynonenal, and deposits of active iron—hallmarks of a ferroptosis signature. Transcriptomic analysis of independent cohorts confirmed that in some patients (up to 30%), the downregulation of ferroptosis defense and glutathione module genes (GPX4, FSP1, GCH1, GSH synthesis genes) is combined with increased expression of PUFA incorporation genes (ACSL4, LPCAT3, etc.), i.e., with a simultaneous increase in the substrate for peroxidation and a decrease in antioxidant capacity (30). This combination may explain the increased sensitivity of hepatocytes to ferroptotic death in some patients.

The critical role of GPX4 in preventing hepatocyte ferroptosis and the possibility of its functional compensation by vitamin E have been clearly demonstrated in experimental models. Hepatocyte-specific inactivation of *Gpx4* in mice leads to massive hepatocyte destruction and death within 48 hours after birth. Adding vitamin E (500 IU/kg) to the mother’s diet completely prevents mortality and hepatocellular degeneration, and such mice are born in the expected Mendelian ratio. However, when these mice are switched to a vitamin E-deficient diet at 6 weeks of age, they develop delayed death against a background of accumulation of lipid peroxidation products (thiobarbituric acid reactive substances (TBARS)). The protective action of vitamin E is based on its ability to break the chains of lipid peroxidation (radical-scavenging, chain-breaking antioxidant), which functionally compensates for the absence of GPX4 (143). These preclinical data form a rational basis for the concept of “antiferroptotic therapy” and offer a mechanistic explanation for the clinical efficacy of vitamin E in some patients with MASH. In particular, in the PIVENS study, the use of vitamin E at a dose of 800 IU/day in patients without diabetes led to a significant improvement in the histological picture of steatohepatitis (43% in the treatment group versus 19% in the placebo group, P=0.001) (144), which was subsequently confirmed in meta-analyses (145). However, the clinical effect of vitamin E cannot be attributed solely to the suppression of ferroptosis. More likely, it reflects a combination of antioxidant, membrane-stabilizing, anti-inflammatory, and potentially anti-ferroptotic effects. Therefore, vitamin E should be described as a clinically available lipophilic radical scavenger with a possible anti-ferroptotic component, rather than as a specific inhibitor of ferroptosis.

Heme oxygenase-1 (HO-1), encoded by the *HMOX1* gene, occupies a special place. On the one hand, HO-1 catalyzes the breakdown of heme into biliverdin, CO, and Fe²^+^. Biliverdin (which rapidly converts to bilirubin) and CO possess antioxidant and anti-inflammatory properties, reducing oxidative damage (146–148). On the other hand, depending on the cellular context, the released free iron (Fe²^+^) can contribute to a pro-ferroptosis signal by catalyzing the generation of hydroxyl radicals in the Fenton reaction and promoting lipid peroxidation (149, 150). Released Fe²^+^ — if its concentration exceeds the buffering capacity of iron storage systems (in particular, ferritin), which occurs, for example, during excessive ferritinophagy (151) or when antioxidant mechanisms such as the GPX4/GSH axis are compromised (152) — can replenish the labile iron pool and, via the Fenton reaction, enhance lipid peroxidation and ferroptosis.

Despite the potentially pro-ferroptotic effect of Fe²^+^ release upon excessive HMOX1 activation (153), in most MASLD/MASH models, evidence favors the protective role of moderate NRF2/HO-1 activation (146, 154, 155), likely due to the concomitant induction of ferritin, antioxidant systems (146, 156), maintenance of mitochondrial homeostasis (157), and suppression of the inflammatory response (158). Therefore, HMOX1 should be viewed not as an unambiguous anti-ferroptotic factor in all biological contexts, but as a context-dependent regulator of the ferroptosis threshold (153), whose protective effect in MASLD/MASH is realized by preserving the cell’s ability to buffer released iron and activate antioxidant programs.

In a study of MASLD datasets, the *HMOX1* gene was identified as a key gene associated with ferroptosis. Moreover, *HMOX1* expression levels were reduced in the liver tissues of patients, a finding confirmed in an experiment using mice with induced steatosis (154). Pharmacological induction of *HMOX1* by hemin in a MASLD model induced by a methionine-choline-deficient diet in mice suppressed ferroptosis, which was accompanied by a decrease in Fe²^+^, MDA, and ROS levels, restoration of GSH and GPX4, and normalization of mitochondrial structure (154). In this study, the protective effect was mediated by inhibition of the janus kinase/signal transducer and activator of transcription (JAK/STAT) pathway. Activation of the NRF2/HO-1 signaling pathway by the natural compound echinacoside also effectively suppressed ferroptosis and reduced steatosis, inflammation, and fibrosis in both a cellular model (HepG2 cell line treated with oleic/palmitic acids) and in *db/db* mice (155). Taken together, these pharmacological data indicate that increased expression of *HMOX1* or activity of HO-1, regardless of the specific activation mechanism, is associated with a reduction in MASLD manifestations.

The most compelling evidence has been obtained in experiments with genetic *Hmox1* deletion. Hepatocyte-specific HO-1 knockout (HO-1^HEPKO^) in mice on a high-fat or MCD diet leads to a significant exacerbation of steatohepatitis: steatosis, ballooning degeneration, and necroinflammation, accompanied by pronounced mitochondrial dysfunction—disruption of mitochondrial dynamics (shift toward fragmentation), a drop in membrane potential, reduced ATP production, and damage to mitochondrial DNA (157). This provides direct genetic evidence that the absence of *Hmox1* in hepatocytes accelerates disease progression. Notably, the protective role of HO-1 is not limited to hepatocytes. In mice with selective *Hmox1* deletion in myeloid cells (*LysM-Cre : Hmox1^flfl^*) in a NASH model induced by a methionine-choline-deficient (MCD) diet for 3 weeks, more pronounced hepatic steatosis and significantly more severe pericellular fibrosis were observed compared to control animals (158). In the same mice, in a model of cholestatic injury induced by bile duct ligation, pro-inflammatory M1-like macrophage polarization was observed, along with a significant increase in ductular reaction and liver fibrosis compared to control animals (158). These results underscore that HO-1 in Kupffer cells and infiltrating macrophages is essential for limiting the inflammatory and fibrogenic response.

Thus, in the pathophysiological context of MASLD/MASH, HO-1 implements its protective program through several complementary mechanisms, specifically the generation of potent antioxidants, the maintenance of mitochondrial homeostasis in hepatocytes, and the promotion of an anti-inflammatory, M2-like phenotype of hepatic macrophages, which limits fibrogenesis. Collectively, these mechanisms form a robust barrier against ferroptotic cell death and slow the progression of steatohepatitis. A summary of the molecular pathways of ferroptosis, their key components, the direction of changes in MASLD/MASH, and the level of evidence is presented in **Table 1**.

**Table 1:** Molecular modules of ferroptosis in the liver in MASLD/MASH: key genes and proteins, direction of changes, and evidence base.

Module	Gene/protein	Function in ferroptosis execution	Change in MASLD/MASH	References
Iron (accumulation of labile Fe²^+^)	TFR1 (*TFRC*)	import of transferrin-bound iron	↑ expression	(27, 29)
	DMT1 (*SLC11A2*)	import of Fe²^+^	↑ expression; may contribute to expansion of the labile Fe²^+^ pool	(27, 29)
	ZIP14 (*SLC39A14*)	uptake of non-transferrin-bound iron	↑; genetic deletion suppresses ferroptosis and fibrosis	(27)
	Ferroportin/FPN1 (*SLC40A1*)	the only iron exporter	↓ mRNA	(27, 29)
	Ferritin (*FTH1, FTL*)	iron storage	iron storage; upon NCOA4-dependent ferritinophagy, ferritin degradation releases Fe²^+^	(27)
	*NCOA4*	ferritinophagy receptor, releases Fe²^+^	↑ ferritinophagy activity; expression change is context-dependent	(27, 63, 64)
	FXN (frataxin)	Fe–S cluster biogenesis, mitochondrial iron handling	↓ in early MASH; deficiency impairs Fe–S cluster biogenesis, increasing catalytically active iron	(124, 125, 167)
Lipid (PUFA-phospholipids — peroxidation substrate)	*ACSL4*	activation of PUFAs (AA, AdA) → PUFA-CoA	↑ mRNA and protein; correlation with steatosis; effect is context-dependent and not reproduced in all dietary models	(28, 29, 75, 76)
	*LPCAT3*	incorporation of PUFAs into sn-2 of PE (PUFA-PE)	dysregulation/context-dependent change; role is dual	(29, 80, 81)
	*FADS1/FADS2, ELOVL5*	biosynthesis of AA/AdA from linoleic acid	altered desaturase/elongase activity; ↓ D5D/FADS1 with MASLD progression changes the long-chain PUFA pool and membrane phospholipid composition	(78–84)
	*FAR1, GNPAT, AGPS*	peroxisomal synthesis of ether PUFA lipids	pro-/anti-ferroptotic balance; FAR1/AGPS in MASLD/MASH are limitedly validated; Gnpat ↓ in steatohepatitis is associated with deficiency of protective plasmalogens	(87–91)
Oxidative (ROS generation, peroxidation initiation)	*ALOX15* + PEBP1	enzymatic oxidation of PUFA-PE → 15-HpETE-PE	may initiate enzymatic oxidation of PUFA-PE, especially under GPX4/GSH deficiency	(68, 72, 95)
	*POR, CYB5R1*	electron transfer → H_2_O_2_ → •OH (Fenton reaction), sustaining ROS formation and iron-dependent lipid peroxidation	experimentally involved in ferroptosis; contribution in MASLD/MASH requires further validation	(100–102)
	*NOX1/NOX2; NOX4*	NADPH oxidases: O_2_^-^ (NOX1/2), H_2_O_2_ (NOX4)	NOX1/2 ↑ (pro-fibrogenic); NOX4 is dual, context-dependent	(97–99, 104–107)
	Mitochondrial ROS	electron leakage → O_2_^-^ → H_2_O_2_	↑ under lipotoxicity	(106, 107)
Antioxidant (protection; ↓ in ferroptosis-high MASH)	Xc^-^ system: *SLC7A11/SLC3A2*	cystine import → cysteine → GSH	dysregulated; ↓ or functional deficiency in ferroptosis-high subgroup; compensatory induction possible upon NRF2/ATF4 activation	(141, 142, 159)
	*GPX4*	detoxification of PL-OOH → PL-OH (GSH-dependent)	↓ expression/activity in ferroptosis-high subgroup; not universal for all MASLD/MASH	(28, 29, 96, 143)
	7-DHC/DHCR7	conversion of the endogenous radical-trapping sterol 7-DHC to cholesterol	context-dependent; DHCR7 inhibition raises 7-DHC and confers ferroptosis resistance; not yet measured in MASLD/MASH	(92–94)
	FSP1 *(AIFM2)/*CoQ10	GPX4-independent protection	↓ in some patients	(30)
	GCH1/BH4	GPX4-independent protection (tetrahydrobiopterin)	↓ in some patients	(30)
	NRF2 (*NFE2L2*)/HO-1 (*HMOX1*)	antioxidant program, Fe²^+^ buffering	context-dependent; moderate NRF2/HMOX1 activation is predominantly protective, but excess free Fe²^+^ may enhance ferroptosis	(146, 153–158)
Calcium/membrane execution	SERCA	ER Ca²^+^ refilling	inhibited by 4-HNE adducts and PKCδ → ER Ca²^+^ depletion	(126–128)
	MCU	mitochondrial Ca²^+^ uptake	↑ (autophagy-dependent); MCU–ACSL4 interaction promotes ferroptosis; hepatic knockdown attenuates MASH	(137, 138)
	NINJ1/ESCRT-III	terminal PM rupture vs. repair	determine death kinetics and immunogenicity	(132, 135)

↑/↓ denote the predominant direction of changes in expression, activity, or functional contribution according to MASLD/MASH studies and experimental models; these changes should not be interpreted as universal for all patients or all cell types. Gene symbols are italicized; proteins, protein complexes, and metabolites are in plain font. AA, arachidonic acid; AdA, adrenic acid; PE, phosphatidylethanolamine; PUFA, polyunsaturated fatty acids; PL-OOH, phospholipid hydroperoxides; PL-OH, reduced phospholipid alcohols; GSH, reduced glutathione; ROS, reactive oxygen species; LIP, labile iron pool; RES, reticuloendothelial system; HSC, hepatic stellate cells.

## 5 Transcriptomic and molecular changes in key genes

### 5.1 Modular architecture of the ferroptosis signature

To interpret ferroptosis in MASLD, it is more productive to analyze not individual genes but functional modules that reflect key biochemical stages of the process: iron metabolism, PUFA-mediated membrane remodeling, enzymatic and non-enzymatic lipid peroxidation, as well as antioxidant defense (27–30, 54–56, 60–62). This approach avoids overinterpretation of isolated changes in expression and better aligns with the modern concept of ferroptosis as a metabolically driven cellular state rather than a linear “gene pathway.” Empirically, it is precisely this modular approach that allows for the identification of patient subgroups with varying ferroptosis susceptibility.

Recent transcriptomic studies in MASLD, including analysis of the public datasets GSE130970, GSE135251, and GSE126848 using machine learning methods (Gaussian Mixture Modeling and gene set variation analysis, GSVA) (30), confirm the existence of at least four interconnected transcriptomic clusters reflecting key processes. The first cluster includes ferroptosis-protective genes (*GPX4, FSP1, GCH1*). The second cluster consists of genes involved in glutathione synthesis and regeneration, such as *SLC7A11, GCLC*, and *GSR*. The third cluster is “PUFA-mediated membrane remodeling.” This includes the genes *ACSL4, LPCAT3, FADS1/2*, and *ELOVL5*. Finally, the fourth block consists of iron metabolism genes, such as *TFRC, FTH1, FTL, SLC40A1, HMOX1*, and *NCOA4*. These blocks may be differentially activated in different patients, determining individual susceptibility to ferroptosis and the potential efficacy of anti-ferroptotic therapy.

It is crucial to note that the direction of change in the expression of these genes is not a direct equivalent of ferroptosis activity. Functional inference requires the integration of transcriptomics with protein levels, enzyme activity, quantitative lipidomics of oxidized phospholipids, assessment of labile iron, and experimental reversibility of the phenotype upon inhibition of ferroptosis. It is particularly important to account for tissue heterogeneity, because an increase or decrease in the expression of a specific gene in a pooled biopsy sample may reflect both changes in the hepatocyte population and a shift in the cellular composition of the tissue due to inflammatory infiltration.

### 5.2 Genes and proteins with potential diagnostic and stratification value

Despite the methodological limitations described, data have already been accumulated for a number of key genes, integrating transcriptomic, proteomic, and functional evidence of their role in the development of ferroptosis susceptibility in MASLD and MASH.

In the category of antioxidant defense genes, reduced tissue expression of *GPX4* is a characteristic feature of a subgroup of MASLD patients exhibiting a biopsy-confirmed “ferroptosis signature” (including accumulation of 4-HNE and redox-active iron) (30, 102). However, a direct association between reduced GPX4 and specifically the progression of MASLD to MASH and the progression of fibrosis has not been established, since this signature is also found in patients with isolated steatosis (30). Concurrently, in some MASLD/MASH patients, a decrease in the expression of not only GPX4 but also other antioxidant defense genes, including FSP1/*AIFM2* and *GCH1*, is observed. This indicates the compromise of parallel GPX4-independent protective pathways and is consistent with the existence of a subgroup of patients with a transcriptomic “ferroptosis signature” (30). The reduction in *SLC7A11* observed in MASLD/MASH indicates a limitation in cystine intake and depletion of the GSH pool, closing the vicious cycle of antioxidant deficiency. As demonstrated in experimental models, loss of *SLC7A11* function leads to cysteine deficiency and a drop in the GSH/oxidized glutathione (GSSG) ratio, which directly contributes to the accumulation of lipid peroxides and the progression of steatohepatitis (159).

Key driver genes include *ACSL4* and *LPCAT3*, whose increased expression facilitates the incorporation of polyunsaturated fatty acids into membrane phospholipids and forms the substrate basis for peroxidation (75, 102, 160). Iron metabolism regulators, such as TFR1 and *FTH1*, may potentially serve as diagnostic markers. Their combined dynamics reflect a shift in intrahepatic iron homeostasis toward an expansion of the labile pool. However, this hypothesis requires validation. Iron metabolism disorders in MASLD/MASH are associated with altered TFR1 expression, which mediates the uptake of transferrin-bound iron by hepatocytes (44, 161, 162). This suggests that TFR1 could be a potential biomarker candidate. However, its clinical validation in this role has not yet been conducted. The role of FTH1 as a potential biomarker for MASLD/MASH also requires further clinical validation, despite existing experimental evidence of its involvement in the pathogenesis of the disease. In MASLD models, altered FTH1 expression in hepatocytes has been demonstrated, suggesting its involvement in iron metabolism disorders and ferroptosis (162). However, studies evaluating its diagnostic accuracy in patients are currently lacking. Reduced expression of *SLC40A1*/FPN1 in the liver is a characteristic feature of iron metabolism disorders in MASLD/MASH (29, 161, 163). This allows FPN1 to be considered a potential biomarker candidate. However, its clinical validation in this role has not yet been conducted.

In addition to direct regulators of iron metabolism, inflammatory and signaling genes reflecting the link between ferroptosis and the immune response are increasingly being included in multigene diagnostic models. First and foremost, this includes *PTGS2* (COX2), a recognized marker of ferroptosis (164, 165), as well as the key pro-inflammatory cytokines IL-6 and IL-1β, which are frequently included in multigene models as markers of the ferroptosis-coupled inflammatory response. Their expression reflects the activation of the “ferroptosis–NF-κB–inflammasome” axis, which has been confirmed in various models of inflammatory diseases (164, 166).

### 5.3 Candidate genes identified by transcriptomic screening methods

In addition to the classical regulatory genes discussed above, modern integrative studies combining bioinformatic analysis of transcriptomic data with experimental validation allow for the identification of new potential biomarkers and therapeutic targets associated with ferroptosis in MASLD/MASH. This approach, based on the analysis of large datasets, allows for the identification of non-obvious molecular links, which are then validated in laboratory models.

In particular, one such study focused on identifying ferroptosis-associated genes differentially expressed in the early stages of MASH. As a result, four new potential biomarkers associated with ferroptosis were identified, namely fatty acid-binding protein 4 (FABP4), frataxin (FXN), sulfhydryl oxidase 1 (QSOX1), and capping actin protein, gelsolin-like (CAPG). The differential expression of these genes was confirmed in both patient biopsies and *in vivo* and *in vitro* models. It is important to note that the functional significance of these genes has been experimentally demonstrated: for example, knockdown of *FABP4* or *CAPG* in hepatocytes led to the suppression of ferroptosis and a reduction in cellular damage (167).

These data are consistent with the results of another independent transcriptomic analysis, in which FABP4 was identified as the primary predictor of progression from simple steatosis to non-alcoholic steatohepatitis, allowing for the identification of a group of patients at high risk of disease progression (168). The role of FABP4 as a marker of progression and a potential therapeutic target is supported by evidence that adipocyte FABP4 is actively secreted in obesity, accumulates in hepatocytes, and contributes to the maintenance of a pro-inflammatory and pro-fibrogenic microenvironment, and its serum levels are significantly elevated in patients with MASLD-associated hepatocellular carcinoma (169).

Frataxin (FXN) deserves special attention. Given its key role in the biogenesis of iron-sulfur clusters and the antioxidant protection of mitochondria, its downregulation in MASH, as demonstrated in this study, appears logical, since MASLD/MASH is characterized by mitochondrial dysfunction and iron homeostasis disorders (170, 171). A direct clinical-transcriptomic study of early-stage MASH showed that the FXN gene is consistently downregulated in patients’ livers and is classified among the key ferroptosis-associated genes with high diagnostic potential (167). Thus, FXN can be considered a potential biomarker of early abnormalities in MASLD/MASH. However, prospective clinical studies measuring serum or liver tissue protein levels are required to definitively confirm its diagnostic value.

Another independent study focused on identifying secreted biomarkers demonstrated that sulfhydryl oxidase 1 (QSOX1) is a ferroptosis-associated gene whose levels increase with the severity of NAFLD, and the QSOX1/interleukin 1 receptor accessory protein (IL1RAP) ratio in plasma demonstrates exceptionally high diagnostic accuracy (area under the receiver operating characteristic curve (AUROC) up to 0.95) (172), opening up prospects for its non-invasive application.

Finally, a study on bioinformatic stratification of NAFLD proposed a number of additional candidates, such as DPP4, sterol carrier protein 2 (SCP2), transferrin (TF) (along with mucin 1 (MUC1) and solute carrier family 1 member 4 (SLC1A4)), which also fit into the biology of ferroptosis: SCP2 controls the intracellular transport of fatty acids and sterols, while TF regulates iron uptake and metabolism in hepatocytes (173). Thus, the range of potential transcriptomic markers of ferroptosis in MASLD/MASH is constantly expanding, and further prospective clinical studies measuring these proteins in serum or liver tissue are necessary to definitively confirm their diagnostic and prognostic value.

## 6 Non-coding RNAs and non-invasive diagnostics

The practical value of transcriptomic modules will only be fully realized after their translation into non-invasive diagnostic tests. Promising areas for such translation include circulating non-coding RNAs (miRNAs, long non-coding RNAs (lncRNAs), circular RNAs (circRNAs)) that regulate ferroptosis genes. In particular, miR-124-3p, miR-761, miR-23a-3p, miR-214-3p, miR-541-3p, miR-129-3p, miR-375, and miR-3200-5p have a documented ability to modulate key ferroptosis genes (*STEAP3, ACSL4, GPX4, SLC7A11, ATF4*) specifically in liver cells (174–187). These molecules can be considered potential candidates for non-invasive diagnosis or therapeutic intervention in liver diseases. However, their role in MASLD/MASH requires targeted investigation. In particular, miR-124-3p suppresses the expression of STEAP3 (six-transmembrane epithelial antigen of the prostate 3), a key regulator of the intracellular iron pool, and thereby inhibits ferroptosis in hepatocytes (180–182). miR-761 directly suppresses the expression of hepcidin (HAMP) and GPX4 in hepatocytes, thereby modulating iron metabolism and antioxidant defense, as confirmed in both *in vitro* and *in vivo* models (183). miR-23a-3p directly suppresses the expression of ACSL4—a key enzyme responsible for the accumulation of oxidizable phospholipids and essential for the execution of ferroptosis. This mechanism has been demonstrated in hepatocellular carcinoma cells, where miR-23a-3p, by inhibiting ACSL4, limits ferroptosis and promotes resistance to sorafenib (174, 182, 184). miR-214-3p acts as a potent pro-ferroptotic factor in liver cells. In hepatocellular carcinoma cells, it suppresses ATF4 and GPX4, while in primary hepatocytes, it activates the transcription of ferroptosis driver genes, including ACSL4, solute carrier family 38 member 1 (SLC38A1), and protein kinase AMP-activated catalytic subunit alpha 2 (PRKAA2) (174, 185, 186). Thus, miR-214-3p represents a key hub integrating signals of oxidative stress and programmed cell death in the liver. miR-541-3p functions as a pro-ferroptotic microRNA in liver cells by directly suppressing the expression of the central antioxidant enzyme GPX4. Binding of miR-541-3p to the 3′-untranslated region of the GPX4 mRNA reduces its levels, contributing to the accumulation of lipid peroxides and the induction of ferroptosis. In hepatocellular carcinoma, the activity of miR-541-3p is regulated by the competing endogenous RNA circIL4R, which, by binding this microRNA, restores GPX4 expression and suppresses ferroptotic cell death (174, 177–179). miR-129-3p directly suppresses the expression of SLC7A11—a key subunit of the cystine/glutamate antiporter of the Xc^-^ system—which leads to a decrease in glutathione synthesis, the accumulation of reactive oxygen species, and the induction of ferroptosis in hepatocytes. This regulatory axis has been experimentally confirmed in a liver model of selenium deficiency in chickens and in chicken hepatoma cell culture (187), and is summarized in a review of ferroptosis regulation by non-coding RNAs (182). miR-375, known for its suppressive effect on SLC7A11 in certain types of cancer (182), may, according to preliminary data, act as a pro-ferroptotic factor in hepatocytes as well (182). However, direct experimental studies in liver models are required to definitively confirm this role in the context of MASLD/MASH. miR-3200-5p functions as a pro-ferroptotic factor in liver cells by directly suppressing the transcription factor ATF4. This leads to a decrease in GPX4 expression and the accumulation of lipid peroxides, which induces ferroptosis. This mechanism has been characterized in detail in hepatocellular carcinoma models, where miR-3200-5p acts as a key effector of the hepatocellular carcinoma up-regulated long non-coding RNA (HULC)/miR-3200-5p/ATF4 axis (174–176).

Thus, the miRNAs discussed are functionally integrated into the iron, lipid, and antioxidant modules of ferroptosis and may serve as a link between tissue transcriptomic signatures and minimally invasive diagnostics. Their stability in the bloodstream, including within exosomes, opens up the prospect of non-invasive monitoring of ferroptosis activity in MASLD/MASH. Single miRNAs have limited specificity. Therefore, the priority is to develop multi-marker panels integrating several miRNA candidates with independent clinical and laboratory indicators. To implement such panels in clinical practice, standardization of detection methods, validation in prospective cohorts, and direct comparison of circulating miRNAs with histological and imaging data are required.

## 7 Cell-specificity and intercellular loops

Ferroptosis in MASLD should be viewed as a tissue-specific process that depends on cell type, disease stage, and the liver microenvironment. Hepatocytes, Kupffer cells, monocyte-derived macrophages, stellate cells, sinusoidal endothelial cells, and adaptive immune cells all undergo ferroptosis, with fundamentally different consequences for disease progression. Therefore, therapeutic targeting of ferroptosis requires not only molecular but also cell-specific stratification. Next, hepatocytes, Kupffer cells, monocyte-derived macrophages, hepatic stellate cells, sinusoidal endothelial cells, and lymphoid populations are examined separately, with an indication for each of them of how robust the evidence base specific to MASLD is.

### 7.1 Hepatocytes

Hepatocytes are the primary target of ferroptosis in MASLD/MASH. The convergence of increased ACSL4/LPCAT3 expression, expansion of the labile iron pool, and weakening of the Xc^-^/GSH/GPX4 axis in hepatocytes results in a pro-ferroptotic phenotype, in which the accumulation of PUFA-PE hydroperoxides exceeds the buffering capacity of antioxidant pathways and culminates in iron-dependent membrane peroxidation (27–30). In NASH models induced by a CDE diet (choline-deficient, ethionine-supplemented), the MCD diet, and the HFD, hepatocyte death is accompanied by all the hallmarks of ferroptosis: accumulation of 4-HNE and MDA, depletion of GSH, decreased GPX4, and reversibility of damage upon treatment with liproxstatin-1, ferrostatin-1, or deferoxamine (27–30, 96, 154, 157).

A fundamental feature of hepatocyte ferroptosis is its immunogenic nature. Unlike apoptosis, in which the cell contents are isolated into apoptotic bodies, ferroptosis is accompanied by early destabilization of the plasma membrane and the release of a whole spectrum of DAMPs, such as HMGB1, ATP, mitochondrial DNA, oxidized phospholipids (oxPLs), and products of non-enzymatic lipid peroxidation (e.g., 4-HNE and MDA adducts) (188–191). These molecules are potent activators of innate immune receptors. It has been shown that HMGB1, released during ferroptosis, binds predominantly to receptor for advanced glycation end-products (RAGE, encoded by the AGER gene), triggering an inflammatory response in macrophages (188). Other DAMPs, such as mtDNA and OxPLs, are capable of activating TLR9 and TLR2/TLR4, respectively, as established in NASH models (190, 191). However, their direct involvement in signal transduction from ferroptotic hepatocytes to Kupffer cells still requires experimental confirmation. Thus, existing data link hepatocyte ferroptosis to RAGE activation, whereas the involvement of TLR2, TLR4, and TLR9 remains probable but unproven. Thus, hepatocellular ferroptosis acts as a primary trigger initiating an inflammatory cascade, which is one of the key pathogenetic features of the transition from steatosis to steatohepatitis (192).

The zonal heterogeneity of hepatocytes may determine the spatial organization of ferroptosis susceptibility in MASLD/MASH. The fundamental metabolic zonation of the liver is manifested by the fact that perivenular (zone 3) hepatocytes function under significantly lower oxygen tension (periportal ~60–65 mm Hg, perivenous ~30–35 mm Hg (193, 194)) and are characterized by high expression of genes for xenobiotic and CYP-dependent metabolism, including *Cyp2e1*, as well as components of the glutathione system (195–198). These same cells are normally under the control of Wnt/β-catenin signaling and possess a reduced capacity for oxidative phosphorylation. These features create conditions for increased production of reactive oxygen species and changes in the intracellular iron pool. Consequently, it is logical to assume that perivenular hepatocytes may exhibit selective vulnerability to ferroptosis in MASH. This spatial organization is accompanied by zonal changes in lipid composition and phospholipid remodeling, including pericentral enrichment with arachidonic acid and disruption of normal phospholipid zonation, which potentially increases the sensitivity of zone 3 hepatocytes to lipid peroxidation (198, 199).

In classical histological descriptions of MASH in adult patients, the pericentral zone (zone 3) of the hepatic acinus invariably appears as the region with the most pronounced changes. It is here that macrovesicular steatosis, ballooning degeneration of hepatocytes, and foci of lobular inflammation are predominantly localized (3, 200, 201). This centrilobular accentuation of damage is a key diagnostic feature in the pathomorphological evaluation of biopsies. Thus, the assertion that ferroptotic events are predominantly localized in zone 3 in MASH should be considered a pathogenetically sound but not yet definitively proven hypothesis. The available data do indeed confirm the involvement of ferroptosis in the initiation of inflammation in steatohepatitis (27, 202), but direct spatial verification of ferroptosis across zones of the hepatic lobule remains limited. Moreover, in an early study by Tsurusaki et al. (192) using a CDE model, initial ferroptotic necrosis was observed predominantly in the periportal region rather than in zone 3, underscoring the need for cautious generalizations and further research in this area.

Periportal (zone 1) hepatocytes, in contrast, possess metabolic features that may contribute to their relative resistance to oxidative damage in the early stages of MASLD. They are characterized by high oxidative phosphorylation intensity, a more developed mitochondrial network, and receive blood with a higher oxygen tension (197). The question of the zonal gradient, however, requires further investigation, as the differences between zone 1 and zone 3 likely represent not an absolute but a relative spatial predisposition to lipotoxicity, lipid peroxidation, and ferroptotic vulnerability during the progression of MASLD/MASH.

### 7.2 Kupffer cells and monocyte-derived macrophages

Kupffer cells and infiltrating monocyte-derived macrophages form the second critical pathway of ferroptotic pathology in MASLD/MASH. Their role is dual: on the one hand, they act as effectors of pro-inflammatory amplification, responding to DAMPs released during hepatocyte ferroptosis and secreting cytokines (IL-6, TNF-α) that can further stimulate ferroptosis (203, 204). On the other hand, macrophages serve as a significant intrahepatic iron reservoir. In MASH, iron overload contributes to their own ferroptotic death, which exacerbates inflammation and fibrosis (203, 205).

Lipid peroxidation products (such as 4-hydroxynonenal and malondialdehyde) and other damage-associated molecules (DAMPs) released during hepatocyte ferroptotic death activate Kupffer cells and pro-inflammatory signaling pathways, including NF-κB and the NLRP3 inflammasome (27, 49, 206). This process may potentially occur via a two-signal model of NLRP3 inflammasome assembly, where ferroptosis products can provide both a priming signal and an activation signal. However, the specific molecular mechanisms linking ferroptosis to the two-stage activation of NLRP3 require further investigation. The first signal (priming) is provided by the activation of innate immune receptors (TLR, NLR) or cytokine receptors (IL-1R, TNFR), which triggers NF-κB-dependent transcriptional induction of NLRP3, pro-IL-1β, and pro-IL-18 (207–209). HMGB1 is discussed as a possible mediator of priming. HMGB1 activates TLR4 and RAGE/AGER and promotes the assembly of the NLRP3 inflammasome (210, 211), along with oxidized phospholipids (oxPL), which are pro-inflammatory DAMP released during ferroptosis (28), and represent likely molecular bridges between ferroptosis and inflammation.

The second signal (assembly/activation) of the NLRP3 inflammasome is triggered by mitochondrial ROS, lysosomal destabilization, K^+^ efflux, and other stress stimuli (208, 212). This leads to the assembly of the NLRP3 inflammasome, activation of caspase-1, and subsequent maturation and secretion of IL-1β and IL-18 (207–209). In some cells, activated caspase-1 cleaves gasdermin D, inducing pyroptotic death, which is accompanied by the release of additional DAMPs, such as HMGB1 (207, 213). This results in a self-perpetuating vicious cycle: hepatocyte ferroptosis supplies molecular stimuli (such as HMGB1 and oxPL) that activate macrophages and the NLRP3 inflammasome, pyroptotic death of macrophages releases a new batch of DAMPs, and the intensification of sterile inflammation, oxidative stress, and lipid peroxidation, in turn, can trigger subsequent waves of hepatocyte ferroptotic vulnerability. It is precisely this cycle that can be considered one of the mechanisms contributing to the establishment of the inflammatory phenotype and the transition from steatosis to steatohepatitis.

Concurrently, macrophages play a key role in local iron homeostasis. Kupffer cells are an important compartment of the reticuloendothelial iron metabolism in the liver, ensuring the phagocytosis of senescent erythrocytes, the processing of heme, and the recycling of iron, including through the involvement of HO-1 (214, 215). In MASLD/MASH, regulation of the hepcidin–ferroportin axis is disrupted: systemic and local inflammation (in particular, via IL-6/STAT3 signaling) stimulates hepcidin overproduction by hepatocytes, which causes accelerated degradation of ferroportin and blocks iron export from macrophages, contributing to its intracellular retention and exacerbating hepatic iron overload (161, 216). This is consistent with the clinically documented pattern of iron deposition in the reticuloendothelial system (RES), which was independently associated with more severe lobular inflammation, marked hepatocyte ballooning, and advanced fibrosis in the large NASH CRN cohort (n=2833; adjusted OR for advanced fibrosis 1.34; 95% CI 1.11–1.62; p=0.003) (65, 66). Iron accumulation in macrophages may increase their own ferroptosis vulnerability. In particular, recent data from the MASH study demonstrate that neutrophil cytosolic factor 1 (NCF1)-dependent regulation of ROS in macrophages disrupts iron homeostasis in Kupffer cells, promotes phospholipid oxidation, and triggers ferroptosis in Kupffer cells via the oxPL/TLR4/hepcidin axis (205).

Macrophage polarization significantly modulates their ferroptosis sensitivity. However, this relationship is context-dependent and cannot be reduced to a simple “M1—more sensitive, M2—more resistant” scheme. On the contrary, in experimental models, pro-inflammatory M1-like macrophages, despite their high pro-oxidant potential, often demonstrate relative resistance to ferroptosis induced by GPX4 inhibition. This resistance is mediated by inducible nitric oxide synthase (iNOS)/NO-dependent suppression of 15-LOX and lipid radical scavenging, which limits the accumulation of phospholipid hydroperoxides (217). In turn, alternately activated M2-like macrophages, lacking high iNOS expression, are significantly more sensitive to GPX4-dependent ferroptotic death in several models (217, 218). The addition of exogenous NO donors can restore the viability of M2 macrophages, confirming the critical role of this radical in protection against ferroptosis. At the same time, the ferroptotic vulnerability of macrophages is a dynamic parameter depending on the tissue microenvironment, iron levels, the activity of the SLC7A11 (also known as xCT)–GSH–GPX4 axis, the intensity of lipid peroxidation, and the state of NRF2/HO-1-dependent stress adaptation (146, 219). In the tumor microenvironment, for example, increased xCT suppresses ferroptosis in tumor-associated macrophages (TAMs), contributing to their pro-tumorigenic polarization (104–107, 157, 158, 219). Myeloid-specific *Hmox1* deficiency in mice in models of cholestatic (bile duct ligation (BDL)) and metabolic (MCD) liver injury leads to a shift in the macrophage response toward a pro-inflammatory M1-like phenotype, accompanied by enhanced ductular reaction, inflammation, and fibrogenesis (158). These data highlight the key cell-specific role of HO-1 in maintaining the anti-inflammatory balance of macrophages and limiting pathological remodeling of liver tissue. This forms the molecular basis for a cautious, cell-specific interpretation of the phenotype associated with *HMOX1*/HO-1.

### 7.3 Hepatic stellate cells

Hepatic stellate cells (HSCs) occupy a position in the ferroptosis landscape that is opposite to that of hepatocytes. Quiescent HSCs are perisinusoidal cells rich in lipid droplets that store retinoids (220). In response to fibrogenic stimuli, quiescent HSCs undergo transdifferentiation into activated, proliferative, and contractile myofibroblasts. This process is accompanied by a dramatic phenotypic shift. The cells begin to actively secrete extracellular matrix components, primarily types I and III collagen, and simultaneously completely lose the large lipid droplets that store retinoids—a characteristic of the quiescent state (221, 222). This transition is accompanied by a metabolic reorganization, specifically increased iron uptake (223, 224), changes in glutathione metabolism, and a shift in the ratio of polyunsaturated to monounsaturated acyl chains (223–226). This metabolic restructuring makes activated, rather than quiescent, cells susceptible to ferroptosis. The induction of ferroptosis in activated HSCs is therefore an antifibrotic, rather than a pathogenic, event.

The mechanistic basis of this phenomenon has been elucidated primarily in models of toxic and cholestatic fibrosis. Ferritinophagy serves as an essential step. The RNA-binding protein ELAVL1 (also known as HuR (Human antigen R)) stabilizes transcripts of the autophagy apparatus and NCOA4-dependent ferritin degradation, and its loss abrogates ferroptosis in activated HSCs (227). In contrast, ZFP36 (also known as Tristetraprolin or TTP) destabilizes *ATG16L1* mRNA, inhibits autophagy, and thereby protects activated HSCs from ferroptotic death. Thus, *ZFP36* inhibition increases their susceptibility (228). Pharmacological evidence follows the same logic. Artemether reduces the severity of carbon tetrachloride (CCl_4_)-induced fibrosis through p53-dependent induction of HSC ferroptosis (229, 230). Pirfenidone inhibits the expression of glutaminase 1 (GLS1) and serine hydroxymethyltransferase 2 (SHMT2)—key enzymes in glutamine and serine metabolism, respectively—in activated HSCs. The resulting decrease in the intracellular pools of glutamate and glycine, which are necessary for GSH synthesis, leads to the depletion of antioxidant defense and sensitizes HSCs to ferroptosis induced, in particular, by erastin (231), while carvedilol triggers HSC ferroptosis via ATF4/SAT1-dependent depletion of spermidine (232).

Two caveats are important to note regarding MASLD/MASH. First, almost all of the data listed were obtained in models involving tetrachloromethane, bile duct ligation, or cell cultures. Meanwhile, the metabolic environment of the liver in MASH is characterized by iron overload, high availability of polyunsaturated fatty acids, and pre-existing oxidative stress. These conditions are not equivalent to those of the toxic model. There is still little direct evidence that HSC ferroptosis limits fibrogenesis in dietary MASH. Where MASH has been studied directly, the relationship is not simply reciprocal. The selective peroxisome proliferator activated receptor-delta (PPARδ) agonist DN203316, by inhibiting hepatocyte ferroptosis, simultaneously attenuated fibrogenesis in MASH models. This suggests that under conditions of lipotoxic stress caused by excess cholesterol, the contribution of ferroptosis to fibrosis is primarily mediated indirectly. Ferroptotic hepatocytes release exosomes containing double-stranded DNA, which activates the Stimulator of interferon genes (STING)– TANK-binding kinase 1 (TBK1)– Interferon regulatory factor 3 (IRF3) signaling cascade in hepatic stellate cells (HSCs), inducing the expression of profibrogenic genes. At the same time, the products of ferroptosis stimulate macrophages to produce pro-inflammatory cytokines. Consequently, the antifibrotic effect of the PPARδ agonist is mediated by protecting hepatocytes from ferroptotic death and disrupting the paracrine activation of HSCs and macrophages, rather than by directly affecting HSC survival (233). Second, systemic pharmacology faces a real conflict of objectives here. A non-selective ferroptosis inhibitor that protects hepatocytes is capable of simultaneously preserving activated HSCs and sustaining fibrogenesis, whereas a non-selective inducer may deplete myofibroblasts at the cost of parenchymal damage. This is the most compelling argument in favor of cell-targeted delivery.

### 7.4 Liver sinusoidal endothelial cells

Liver sinusoidal endothelial cells (LSECs) form the fenestrated, discontinuous lining of the hepatic sinusoids and are among the first cell types to undergo phenotypic changes in MASLD (234). Early loss of fenestrations (capillarization) and subsequent deposition of the basement membrane precede the development of fibrosis (234, 235). These structural changes limit the bidirectional exchange of lipoproteins between plasma and hepatocytes, specifically impairing the clearance of triglyceride-rich lipoproteins (234), and simultaneously eliminate the tonic NO-dependent suppression of hepatic stellate cell activation, which is normally provided by healthy LSECs (234, 235). Furthermore, LSECs are professional scavenger cells with high endocytic and iron-binding activity, which ensures their constant contact with oxidized lipoproteins and with the labile iron pool of the sinusoidal compartment. These properties make LSECs intrinsically vulnerable to lipid peroxidation. Direct evidence of their ferroptotic death has now been obtained. During liver transplantation, cold storage induces selective ferroptosis of LSECs, which determines the severity of reperfusion injury and the outcome of transplantation and is prevented by ferrostatin-1 (236). During warm ischemia-reperfusion of the liver, there is a sharp increase in epithelial membrane protein 1 (*EMP1*) expression in LSECs. EMP1 activates the p38 mitogen-activated protein kinase (MAPK) signaling pathway, which triggers ferroptosis in LSECs, manifested by Fe²^+^ accumulation, lipid peroxidation, suppression of GPX4, and upregulation of ACSL4. The LSEC ferroptosis induced in this way directly exacerbates hepatocyte damage, whereas EMP1 knockdown or p38 MAPK inhibition prevents these changes and protects the liver from reperfusion injury (237). In metabolic liver disease, a calcium-dependent pathway is also involved. Cytosolic Ca²^+^ overload causes LSEC defenestration, and its pharmacological inhibition restores fenestration and reduces the severity of experimental steatotic liver disease (139). Data obtained directly from dietary models of MASLD remain the weakest link in this chain. Therefore, LSEC ferroptosis in MASLD is described as presumptive rather than established. Its potential significance, however, is disproportionate to the number of cells involved. Since the endothelium controls both the delivery of oxidizable lipids to hepatocytes and the activation status of stellate cells, ferroptotic loss or dysfunction of even a limited population of LSECs is capable of linking the parenchymal and fibrogenic components of the disease. To test this hypothesis directly, studies are needed that combine three methodological approaches. First, genetic reporters (fluorescent labels) that function exclusively in sinusoidal endothelial cells should be used. Second, spatial transcriptomics must be applied. Third, lipid analysis of the sinusoidal compartment itself is required.

### 7.5 Adaptive and innate lymphoid cells

Lymphocytes are not passive observers of hepatic lipid peroxidation. They belong to the cell types most dependent on GPX4 for survival. Genetic deletion of Gpx4 in T cells induces their ferroptotic death, prevents their expansion following antigen encounter, and abolishes protective anti-infectious immunity. Moreover, this defect is corrected by vitamin E (238). The immunological implication is that any condition that increases the content of polyunsaturated fatty acids in lymphocyte membranes or reduces their antioxidant reserve will predominantly deplete the lymphocyte compartment.

A steatotic liver represents precisely such a condition. In mice with NAFLD, hepatic CD4^+^ lymphocytes—unlike CD8^+^ cells—are selectively lost due to linoleic acid-induced production of mitochondrial reactive oxygen species and lipid peroxidation, and this selective loss accelerates hepatocarcinogenesis (239). Thus, ferroptosis vulnerability varies among different lymphocyte subpopulations, and the overall effect on the liver depends on which specific subpopulation is depleted. The remaining compartment, in turn, becomes pathogenic itself. In MASH, hepatic CXCR6^+^CD8^+^ T cells acquire an autoaggressive, antigen-independent phenotype and kill hepatocytes in a manner that sustains, rather than resolves, tissue damage (240). This same population of CD8^+^PD1^+^ cells does not support but rather disrupts immune surveillance during therapy with checkpoint inhibitors for MASH-associated hepatocellular carcinoma (241). The picture is further complicated by antigen-presenting and innate lymphoid cell populations. In hepatocellular carcinoma associated with steatohepatitis, signaling via T cell immunoglobulin and mucin domain-containing protein (Tim-3) triggers ferroptosis in dendritic cells and thereby impairs antitumor immunity, whereas blocking this pathway restores it (242). In a fibrotic liver, CD36 expression on natural killer (NK) cells increases sharply. This receptor mediates excessive lipid uptake, leading to the accumulation of reactive oxygen species, lipid peroxidation, and ferroptosis of NK cells. As a result, the production of interferon-gamma (IFN-γ), perforin, and granzyme B decreases, and the cytotoxic activity of NK cells against activated hepatic stellate cells (HSCs) declines. Blocking CD36 or pharmacologically inhibiting ferroptosis restores the antifibrotic function of NK cells, and the adoptive transfer of CD36-deficient NK cells significantly attenuates fibrosis in mice. Thus, the lipid-enriched microenvironment, via the CD36–ferroptosis axis, inactivates the endogenous NK-cell-mediated inhibition of fibrogenesis (243).

## 8 Therapeutic prospects

The accumulated data on the molecular modules and cell-specific pathways of ferroptosis form the basis for four major directions in therapeutic development. All of them are united by a common principle: the efficacy of ferroptosis-targeted therapy is likely to be realized only in stratified patient groups and through the use of cell-specific delivery approaches.

Before these areas are examined individually, it is necessary to state a general caveat explicitly. With the sole exception of vitamin E, none of the agents discussed below has been studied in a clinical trial for MASLD/MASH with endpoints related to ferroptosis, and none of them is a selective modulator of ferroptosis in the pharmacological sense. Ferrostatin-1 and liproxstatin-1 are chemical probes with unfavorable pharmacokinetic properties, not drug candidates. Deferoxamine and deferasirox act on the systemic iron pool and are approved for the treatment of transfusion-related iron overload, not metabolic liver disease. Abemaciclib is a CDK4/6 inhibitor, and its activity against ACSL4 represents an off-target effect. Hemin, sodium selenite, and plant-derived NRF2 activators modulate broad stress response programs, of which ferroptosis is only one. Current consensus-based methodological recommendations also lead to this conclusion. Since none of the available markers or inhibitors are fully specific, the therapeutic effect attributed to ferroptosis must be confirmed by a combination of orthogonal assessment methods and both pharmacological and genetic interventions (244). The strategies outlined below should therefore be considered mechanistic hypotheses supported by preclinical data, rather than a treatment algorithm.

### 8.1 Radical scavengers and ferroptosis inhibitors

The most clinically advanced representative of this group is vitamin E, whose mechanism of action and clinical evidence base—including the PIVENS study (144) and subsequent meta-analyses (145) — are outlined above in the section on antioxidant protection. From a therapeutic standpoint, vitamin E should be positioned as a clinically accessible lipophilic radical scavenger with a possible anti-ferroptotic component, rather than as a specific inhibitor of ferroptosis. The histological improvement observed in patients without diabetes was not shown to be specifically dependent on the suppression of ferroptosis.

Selective ferroptosis inhibitors—ferrostatin-1 and liproxstatin-1—consistently reduce the severity of steatohepatitis in preclinical MASLD models in which hepatocyte ferroptosis is the dominant mechanism of injury (27–30, 96). Their clinical translation is limited by pharmacokinetic and toxicological issues. However, they serve as important tools for confirming the functional significance of ferroptosis in a specific model and for selecting molecular patient stratifiers.

A conceptually distinct addition to this class is the endogenous radical scavenger 7-dehydrocholesterol. It does not need to be administered externally. It can accumulate upon inhibition of DHCR7, and this approach protects hepatocytes from ferroptosis in models of acute liver injury (93, 94). Evaluation of this strategy in MASLD/MASH has not yet begun, and it carries an obvious risk, since a persistent increase in 7-DHC mimics the biochemical hallmark of Smith–Lemli–Opitz syndrome (245). It has been experimentally demonstrated that pharmacological inhibition of DHCR7 also leads to an increase in 7-DHC, mimicking the biochemical phenotype of the syndrome (246). Any translational application would therefore require careful control of dosage and tissue specificity.

### 8.2 Iron chelation and correction of iron phenotype

Iron chelators (deferoxamine, deferasirox) reduce the availability of labile Fe²^+^ for Fenton reactions and thereby limit both enzymatic and non-enzymatic peroxidation of PUFA-phospholipids. In preclinical models of steatohepatitis, iron chelation consistently reduces levels of lipid peroxidation markers (MDA), restores GSH, and decreases the severity of steatosis, inflammation, and hepatocyte death (27, 96, 154). The clinical niche for these drugs in MASLD/MASH has not yet been defined. However, patients with a confirmed RES pattern of iron deposition (66) or a transcriptomic signature of an expanded labile iron pool appear to be the most reasonable target subgroup for pilot clinical trials.

### 8.3 Targeting ACSL4 and lipid remodeling

Long-chain acyl-coenzyme A (CoA) synthase 4 (ACSL4) is one of the most well-established ferroptosis-dependent mediators of MASH (see the section on the lipid module of ferroptosis). Pharmacological inhibition of ACSL4 with abemaciclib (a CDK4/6 inhibitor with off-target activity against ACSL4) reduces steatosis, inflammation, and fibrosis in models fed an HFD and MCD (75, 76), however, the effects may involve both ACSL4-dependent and CDK4/6-dependent mechanisms, which limits direct clinical application. The development of selective ACSL4 inhibitors without off-target CDK4/6 activity appears to be a priority. Additional targets of the lipid module include LPCAT3 (with due consideration of its context-dependence), as well as enzymes of the peroxisomal-ester lipid pathway, which require preliminary validation in MASLD/MASH models.

### 8.4 Activators of NRF2/HMOX1 pathway and parallel antioxidant pathways

Pharmacological activation of the NRF2/HO-1 pathway reproducibly exerts a hepatoprotective effect in experimental models of MASLD/MASH. *HMOX1* induction via hemin in an MCD-induced NASH model suppressed ferroptosis: this was accompanied by reduced levels of Fe²^+^, malondialdehyde, and reactive oxygen species, restored glutathione levels and GPX4 activity, and normalized mitochondrial ultrastructure (154). The natural activator of the same pathway, echinacoside, effectively suppressed ferroptosis and attenuated steatosis, inflammation, and fibrosis in both HepG2 hepatocyte cultures and *db/db* mice (155). Given the data on the dual role of HMOX1 depending on the cellular and disease stage context, cell-specific targeting of the NRF2/HMOX1 axis appears more promising than systemic targeting. Parallel antioxidant pathways (FSP1/CoQ10, GCH1/BH4) remain primarily preclinical targets for now. However, their activation could become a strategy for patients with compromised GPX4-dependent protection.

### 8.5 Stratified clinical trial design

The main practical implication of the reviewed material is that ferroptosis is a valid therapeutic target only for a molecularly stratified subgroup of patients with MASLD/MASH. According to estimates, this subgroup accounts for approximately 30–60% of cases, depending on the stratification method and cutoff thresholds (30). This necessitates a shift from “all-population” clinical trial designs for anti-ferroptotic strategies to designs with biomarker-stratified randomization.

Cell-specific stratification forms a mandatory second dimension of the therapeutic design. Systemic ferroptosis inhibitors may protect hepatocytes but simultaneously block the antifibrotic effect of selective ferroptotic death of activated HSCs. Systemic inducers, conversely, are theoretically capable of reducing fibrosis but exacerbating hepatocellular damage. This makes cell-specific delivery (lipid nanoparticles (LNP) with hepatocyte targeting, HSC-specific carriers, nanoparticles with macrophage targeting) a priority area for pharmacological development. Another significant limitation is the dependence of adaptive and innate immune cells on GPX4-mediated protection against ferroptosis. Systemic inhibition of ferroptosis, which protects hepatocytes, can simultaneously preserve the pool of autoaggressive CXCR6^+^CD8^+^ T cells that sustain parenchymal damage in MASH, thereby negating part of the therapeutic benefit. Conversely, systemic induction of ferroptosis, even if it limits fibrogenesis by inducing the death of activated stellate cells, is highly likely to exacerbate the antitumor immune surveillance that is already compromised in MASH, accelerating the ferroptosis of dendritic cells and effector lymphocytes. Consequently, clinical trials of both anti- and pro-ferroptotic interventions should include immunological endpoints from the earliest phases to assess the balance between the desired effect on hepatocytes and the undesirable modulation of hepatic immunity.

Multimarker non-invasive panels combining tissue transcriptomic modules with circulating proteins (FABP4, FXN, QSOX1) and circulating miRNAs (miR-124-3p, miR-23a-3p, miR-214-3p, miR-541-3p, miR-129-3p, miR-3200-5p, etc.), represent the most realistic path for integrating ferroptosis-oriented diagnostics into clinical practice. Their development requires standardization of detection methods, validation in prospective cohorts, and direct comparison with histological and imaging references. Clinical studies of anti-ferroptotic strategies—ranging from vitamin E and natural NRF2/HO-1 activators to selective ACSL4 inhibitors and iron chelators—should be designed from the outset so that the ferroptosis signature is used as a patient selection criterion or as a stratification factor during randomization, rather than as a basis for arbitrary subgrouping after the completion of the study.

## 9 Methodological limitations

Any interpretation of ferroptosis in MASLD/MASH is limited by a number of methodological considerations. First, transcriptomic data are not equivalent to the functional activity of ferroptosis. Changes in expression must be integrated with protein levels, the activity of relevant enzymes, quantitative lipidomics of oxidized phospholipids, assessment of labile Fe²^+^, and the pharmacological or genetic reversibility of the phenotype. Second, transcriptomic data derived from liver biopsies reflect changes in the cellular composition of the tissue. Inflammatory infiltration, an increase in the number of macrophages, or activation of stellate cells can mimic or mask hepatocyte-specific shifts. Therefore, future studies should include deconvolution of bulk data, as well as snRNA-seq and spatial transcriptomics methods. Third, experimental results are highly dependent on diet type, genetic background, sex, and age of the animals (in animal studies) and donors (in human studies), gut microbiota composition, and housing conditions, which necessitates the mandatory replication of key results in at least two independent dietary models. Fourth, cell-specific approaches need to be developed for a more accurate interpretation of the results. Fifth, there are virtually no prospective clinical studies in which circulating biomarkers (including multi-marker miRNA panels) have been correlated with histological and imaging endpoints in the same patients with MASLD/MASH in a longitudinal cohort. Sixth, some of the mechanisms included in this review are based on data obtained outside the MASLD/MASH context. The 7-dehydrocholesterol pathway has been characterized in tumor cells and models of acute liver injury, but not in dietary steatohepatitis. Ferroptosis of stellate and sinusoidal endothelial cells has been demonstrated primarily in toxic, cholestatic, and ischemic-reperfusion models. Calcium dynamics during hepatic ferroptosis have not been measured in human tissues.

## 10 Conclusion and perspectives

Thus, ferroptosis represents a distinct, metabolically driven type of regulated cell death that integrates three central pathogenic mechanisms: iron dysmetabolism, accumulation of oxidizable PUFA phospholipids, and depletion of antioxidant defenses. The molecular architecture of ferroptosis in the liver in MASLD corresponds to four functional modules. The lipid module includes PUFA-mediated membrane remodeling (*ACSL4*, *LPCAT3, FADS1/2, ELOVL5*) along with the peroxisomal ether lipid pathway. The iron module includes TFR1, DMT1/*SLC11A2*, *SLC39A14*, ferritin (*FTH1/FTL*), FPN1/*SLC40A1*, and *NCOA4*-dependent ferritinophagy. The oxidative module includes the lipoxygenases ALOX12/15, POR/CYB5R1, the NOX system, and mitochondrial reactive oxygen species. The antioxidant module includes the Xc^-^/*SLC7A11*/GSH/GPX4 axis, FSP1/CoQ10, GCH1/BH4, NRF2/HMOX1, and the recently described 7-dehydrocholesterol brake. This modular representation of the disease is not merely descriptive. Each module contains measurable biomarkers and potential pharmacological targets, and the outcome for the hepatocyte is determined by an imbalance between the modules, rather than by the behavior of any single gene.

It is important to note that when analyzing data on ferroptosis in MASLD, the characteristics of various types of liver cells must be taken into account. Hepatocyte damage is accompanied by the release of DAMPs and the activation of NLRP3- and cyclic GMP-AMP synthase–stimulator of interferon genes (cGAS–STING)-dependent inflammatory pathways. In Kupffer cells and monocyte-derived macrophages, ferroptosis amplifies inflammation and creates a local iron reservoir that sustains a pro-ferroptotic environment. In activated stellate cells, it can limit fibrogenesis. However, this anti-fibrotic effect is offset by massive hepatocyte damage. In adaptive immune cell populations, ferroptosis modulates immune surveillance and may contribute to the progression of MASH to hepatocellular carcinoma. The data discussed above expand this cell-specific picture in two ways. In sinusoidal endothelial cells, ferroptosis and calcium-dependent defenestration disrupt the function of the vascular interface, which controls both the delivery of lipids to hepatocytes and the quiescence of stellate cells. In lymphoid and dendritic cells, dependence on GPX4 renders the immune compartment intrinsically vulnerable to the same lipid peroxidation that damages the parenchyma. As a result, surveillance and repair functions are lost, while autoaggressive effector populations are preserved.

Three sets of issues will shape the immediate research agenda. The first relates to diagnostics. Since no single marker is specific to ferroptosis, the field requires a consensus minimum set of criteria for human liver tissue that combines quantitative lipidomics of oxidized phospholipid species with measurement of the labile pool of ferrous iron, protein assessment of GPX4, FSP1, and ACSL4, and a demonstration of the pharmacological or genetic reversibility of the phenotype (244). Until such a set is agreed upon and applied to the same biopsy specimens, estimates of the size of the subgroup with high ferroptosis activity (currently ranging from approximately 30% to 60% depending on the method) will reflect the methodology rather than the biology.

The second set of questions is cell-biological in nature and concerns the directionality of the effect. The same molecular event has opposite consequences in different liver cells: it is damaging in hepatocytes and sinusoidal endothelial cells, enhances inflammation in macrophages, has an antifibrotic effect in activated stellate cells, and is immunosuppressive in lymphocytes. No systemically administered modulator is capable of meeting all these requirements simultaneously. There is a recurring debate over whether ferroptosis should be inhibited or induced in chronic liver diseases. One of the key questions is which cell type, at which stage of the disease, and via which delivery system should be targeted. Progress in this area depends not so much on the discovery of new targets as on cell-targeted pharmacology, including lipid nanoparticles with tropism for hepatocytes or stellate cells and conjugates that utilize scavenger receptors on sinusoidal endothelial cells and Kupffer cells.

A third set of questions concerns translation, and there are several unresolved contradictions here. The protective effect of ACSL4 deletion in MASH depends on the diet and model. Whether HMOX1 induction is protective or exacerbating is determined by the cell’s ability to buffer released iron. Whether the reduction in hepatic GPX4 reflects disease progression or merely accompanies steatosis remains unclear, as the same signature is observed in patients without steatohepatitis. Finally, the recently described 7-dehydrocholesterol axis is supported by an evidence base, one component of which is currently the subject of an editorial note. Acknowledging these discrepancies is not a weakness of the field, but rather a prerequisite for planning studies capable of resolving them.

Thus, ferroptosis provides a coherent molecular explanation of how disturbances in iron, lipid, and redox balance converge at hepatocyte membranes in MASLD, and offers a set of measurable and potentially pharmacologically targetable nodes. However, its clinical value is unlikely to be realized through interventions targeting the entire patient population. It can only be realized by identifying a subgroup of patients in whom this pathway is truly active, taking into account the opposing roles of ferroptosis in different cells of the hepatic lobule, and testing anti-ferroptotic agents against endpoints that specifically measure the mechanism they are designed to target.
